# The *Phytophthora parasitica* effector AVH195 interacts with ATG8, attenuates host autophagy, and promotes biotrophic infection

**DOI:** 10.1186/s12915-024-01899-w

**Published:** 2024-04-29

**Authors:** Serena Testi, Marie-Line Kuhn, Valérie Allasia, Pascaline Auroy, Fantao Kong, Gilles Peltier, Sophie Pagnotta, Julie Cazareth, Harald Keller, Franck Panabières

**Affiliations:** 1 Université Côte d’Azur, INRAE, CNRS, Institut Sophia Agrobiotech, 06903 Sophia Antipolis, France; 2 Aix Marseille Université, CEA, CNRS, Institut de Biosciences et Biotechnologies Aix-Marseille, CEA Cadarache, 13108 Saint Paul-Lez-Durance, France; 3 Université Côte d’Azur, Centre Commun de Microscopie Appliquée, 06108 Nice, France; 4 Université Côte d’Azur, CNRS, Institut de Pharmacologie Moléculaire et Cellulaire, 06903 Sophia Antipolis, France; 5 Present Address: Station Biologique de Roscoff, UMR8227 LBI2M, CNRS-Sorbonne Unversité, 29680 Roscoff, France; 6 Present address: School of Bioengineering, Dalian University of Technology, Dalian, 116024 China

**Keywords:** Oomycete, Effector, Biotrophy, Autophagy, Vacuoles

## Abstract

**Background:**

Plant pathogens secrete effector proteins into host cells to suppress immune responses and manipulate fundamental cellular processes. One of these processes is autophagy, an essential recycling mechanism in eukaryotic cells that coordinates the turnover of cellular components and contributes to the decision on cell death or survival.

**Results:**

We report the characterization of AVH195, an effector from the broad-spectrum oomycete plant pathogen, *Phytophthora parasitica*. We show that *P. parasitica* expresses *AVH195* during the biotrophic phase of plant infection, i.e., the initial phase in which host cells are maintained alive. In tobacco, the effector prevents the initiation of cell death, which is caused by two pathogen-derived effectors and the proapoptotic BAX protein. AVH195 associates with the plant vacuolar membrane system and interacts with Autophagy-related protein 8 (ATG8) isoforms/paralogs. When expressed in cells from the green alga, *Chlamydomonas reinhardtii*, the effector delays vacuolar fusion and cargo turnover upon stimulation of autophagy, but does not affect algal viability. In *Arabidopsis thaliana*, AVH195 delays the turnover of ATG8 from endomembranes and promotes plant susceptibility to *P. parasitica* and the obligate biotrophic oomycete pathogen *Hyaloperonospora arabidopsidis*.

**Conclusions:**

Taken together, our observations suggest that AVH195 targets ATG8 to attenuate autophagy and prevent associated host cell death, thereby favoring biotrophy during the early stages of the infection process.

**Supplementary Information:**

The online version contains supplementary material available at 10.1186/s12915-024-01899-w.

## Background

Oomycetes have modeled agriculture since the nineteenth century, and plant protection strategies have still to cope with emerging and re-emerging pathogens belonging to this deep assemblage of filamentous eukaryotic microbes [1]. Oomycetes share with other plant pathogens the ability to possess hundreds of virulence genes encoding effectors that are delivered into plant cells to modulate host cellular processes, defeat defense mechanisms, and ultimately promote infection [2]. Effectors from the RxLR class are by far the most prominent functionally characterized group in oomycetes. They are generally small (10–25 kDa) proteins without any obvious functional domain, although both predictive and experimental approaches suggest that many of them would adopt common structures despite substantial sequence diversity [3]. Deciphering precisely their mode of action requires combined approaches, including expression profiling, comparative analysis of sequences and subsequent three-dimensional structures, as well as the characterization of their molecular targets in the host [4]. The development of combined approaches led to the uncovering of the nature of plant functions targeted by effectors and to a better understanding of the contribution of these functions to the outcome of plant-pathogen interactions [5].

In recent years, autophagy has emerged as a cellular mechanism in the host that is frequently targeted by pathogen effectors. Autophagy is an essential recycling mechanism in eukaryotic cells that coordinates the orderly degradation and recycling of cellular components, ensuring the availability of metabolic building blocks and the repair and turnover of damaged cellular components. Autophagy is associated with various signaling pathways that lead to biotic and abiotic stress responses, to senescence, and cell death [6–9]. The proteins that constitute the core machinery of the mechanism are highly conserved among eukaryotes and are manipulated by effectors from viral [10], bacterial [11], fungal [12], and oomycete pathogens [13]. Effectors may either inhibit or stimulate autophagy, but subversion of this mechanism in the host promotes in all cases disease. Some effectors of viruses or necrotrophic fungi inhibit autophagy [10, 12], whereas effectors of other pathogens stimulate this process, with both manipulations promoting the success of infection by the respective pathogens [11, 13]. More complex is the situation when a single pathogen produces multiple effectors that interfere antagonistically with autophagy in the host cell. The bacterium *Pseudomonas syringae* pv *tomato* (*Pst*) secretes at least 4 effector proteins that target autophagy with different outcomes for the mechanism, but promote disease in all cases. The *Pst* effectors HrpZ and HopM1 stimulate autophagy, whereas AvrPtoB and HopF3 inhibit the process [11, 14]. Intriguingly, both HrpZ and HopF3 target ATG8 and promote bacterial infection despite their opposite effects on autophagy [11]. These observations seem to indicate that the precisely timed secretion of different effectors targeting the same protein is essential for fine-tuning host cell autophagy to benefit infection.

In plants, the autophagy-related protein ATG8 appears to be a key target for the manipulation of autophagy during infection. This central hub serves as a docking platform for selective autophagy adaptors and receptors that interact via ATG8-interacting motifs (AIMs) with the protein. To date, only one effector from an oomycete pathogen has been described to interfere with autophagy and, in particular, to target ATG8. PexRD54 from the potato late blight pathogen, *Phytophthora infestans*, specifically targets the isoform ATG8CL in potato and interferes with the formation of a complex between the protein and Joka2, an autophagic cargo receptor. The interference stimulates autophagy, and it has been suggested that this favors the elimination of plant defense-related compounds and/or to provide the pathogen with degradation products [13].

Here, we report the characterization of the effector AVH195 from *Phytophthora parasitica* Dastur (syn *P. nicotianae* Breda de Haan), a soilborne oomycete pathogen that has been reported on 255 plant genera in 90 families [15]. Like most *Phytophthora* species, *P. parasitica* has a hemi-biotrophic lifestyle, meaning that the microbe invades host tissues initially as a biotrophic pathogen, before it switches to necrotrophy and kills the host. The biotrophic phase enables the pathogen to suppress rejection and to settle stably in the host tissues, while the necrotrophic phase promotes a rapid increase in biomass and spread [16]. Here we present data indicating an essential role of AVH195 in impairing host cell autophagy and suggesting that the effector thereby supports the biotrophic phase of the infection process.

## Results

### AVH195 transcripts accumulate during biotrophic infection

**Fig. 1:**
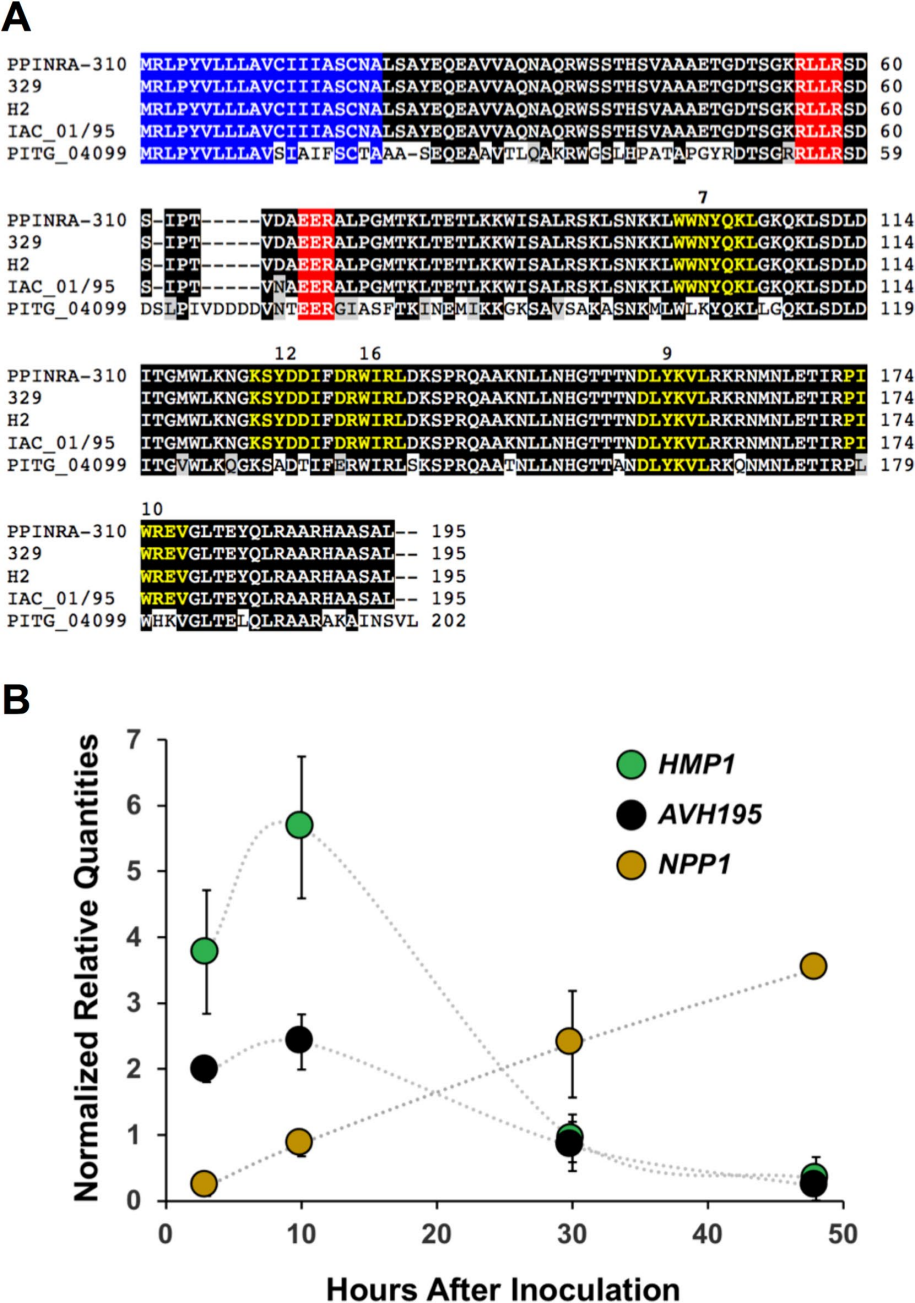
Conservation of AVH195 among *Phytophthora parasitica* isolates and expression during infection*.* **A** *P. parasitica* isolates from different hosts and geographic origins produce highly conserved variants of AVH195 with 99-100% identity at the amino acid level. The Australian isolate INRA_PP310 has a broad host range, while the isolates 329, H2, and IAC_01/95 from Greece, French Polynesia, and Brazil, respectively, are specialized for tobacco, vanilla, and citrus. The closest relative in other *Phytophthora* species is PITG_04099 from *P. infestans*, which shows 67% identity to the *P. parasitica* protein. See Additional file 1: Table S1 for a detailed overview of 20 different *P. parasitica* isolates. Shown are the signal peptide sequences for secretion in blue, the RxLR-EER motifs in red, and the AIMs in yellow. Numbers above the AIMs indicate the iLIR PSSM values. Amino acid alignments were performed with Clustal Omega and edited with Boxshade. Shading indicates blocks of identical (black) or similar (grey) amino acids. **B** Expression of *AVH195* by *P. parasitica* (isolate PPINRA-310) during infection of Arabidopsis correlates with the biotrophic phase of oomycete life cycle. Relative *AVH195* mRNA levels (black) were determined at different timepoints of infection by quantitative RT-PCR in *P. parasitica-*inoculated *A. thaliana*. The expression profiles of *HMP1* (green) and *NPP1* (brown) are markers for the biotrophic and necrotrophic stages of infection, respectively. Relative transcript quantities were normalized with transcripts from the *UBC* and *WS41* reference genes. Presented are the means (± SD) from 3 biological replicates

*AVH195* was characterized as an RxLR effector-encoding sequence in an Expressed Sequence Tag (EST) library generated from plant tissues infected with *P. parasitica* isolate PPINRA-310 [17]. The entire transcript encodes a 195-amino acid (aa) secreted protein that possesses a 20-aa signal peptide, the canonical RxLR-EER motif, and a 125-amino acid predicted effector (Pfam PF16810) domain. The effector domain does not have other subdomains or motifs that allowed speculation about its function, except for 5 potential Atg8-interacting motifs (AIMs), also referred to as LC3-interacting regions (LIRs) [18]. AIMs were detected in the AVH195 sequence by comparison with a collection of LIR motif-containing proteins (LIRCPs) from various organisms that are compiled at the iLIR autophagy database (https://ilir.warwick.ac.uk/index.php). The AIMs of LIRCP are defined by a position-specific scoring matrix (PSSM), which is derived from the alignment of experimentally verified AIM/LIR motifs [19]. The values for AVH195-AIMs ranged from 7 to 16, with 3 AIMs having a PSSM value of ≥ 10 (Fig. 1A). Data mining and in-house analyses of the genomes from 20 *P. parasitica* isolates revealed that the *AVH195* sequence is highly conserved among them, showing ≥ 98.5% identity at the nucleotide level, independent of host specificities and geographical origins of the isolates (Fig. 1A; Additional file 1: Table S1). BLAST searches did not reveal any ortholog in other *Phytophthora* species, except the 202-aa sequence PITG_04099 from the potato late blight pathogen, *P. infestans*, which presents 66.7% identity with AVH195 (Fig. 1A).

*AVH195* was not found in transcriptome data from non-infectious structures of the pathogen, and we therefore hypothesized that the RxLR effector contributes to the infection process. To confirm this hypothesis, we examined the expression of *AVH195* in roots of Arabidopsis plantlets at 3, 10, 30, and 48 h post inoculation (hpi) with zoospores from *P. parasitica*. This time course was previously shown to describe the biotrophic stage of the infection cycle and includes the invasive growth step that corresponds to the switch from biotrophy to necrotrophy at 30 hpi [20]. *AVH195* mRNA was not detectable in non-invasive stages of the *P. parasitica* life cycle, like motile zoospores, germinated cysts, or mycelial cultures. By contrast, *AVH195* transcripts accumulated in the early steps of infection, then slowly decreased during later steps, and became barely detectable at 48 hpi (Fig. 1B). To correlate the expression profile with the life cycle of *P. parasitia in planta*, we monitored expression of the genes encoding Haustorium-Specific Membrane Protein 1 (HMP1) and Necrosis-inducing *Phytophthora* Protein 1 (NPP1), which are considered as markers for the biotrophic and necrotrophic stages of *Phytophthora* infection, respectively [21]. Based on their respective mRNA levels (Fig. 1B), we concluded that *AVH195* transcripts preferentially accumulate during the biotrophic stage of the *P. parasitica* infection cycle.

### AVH195 prevents onset of cell death in plant tissues

**Fig. 2:**
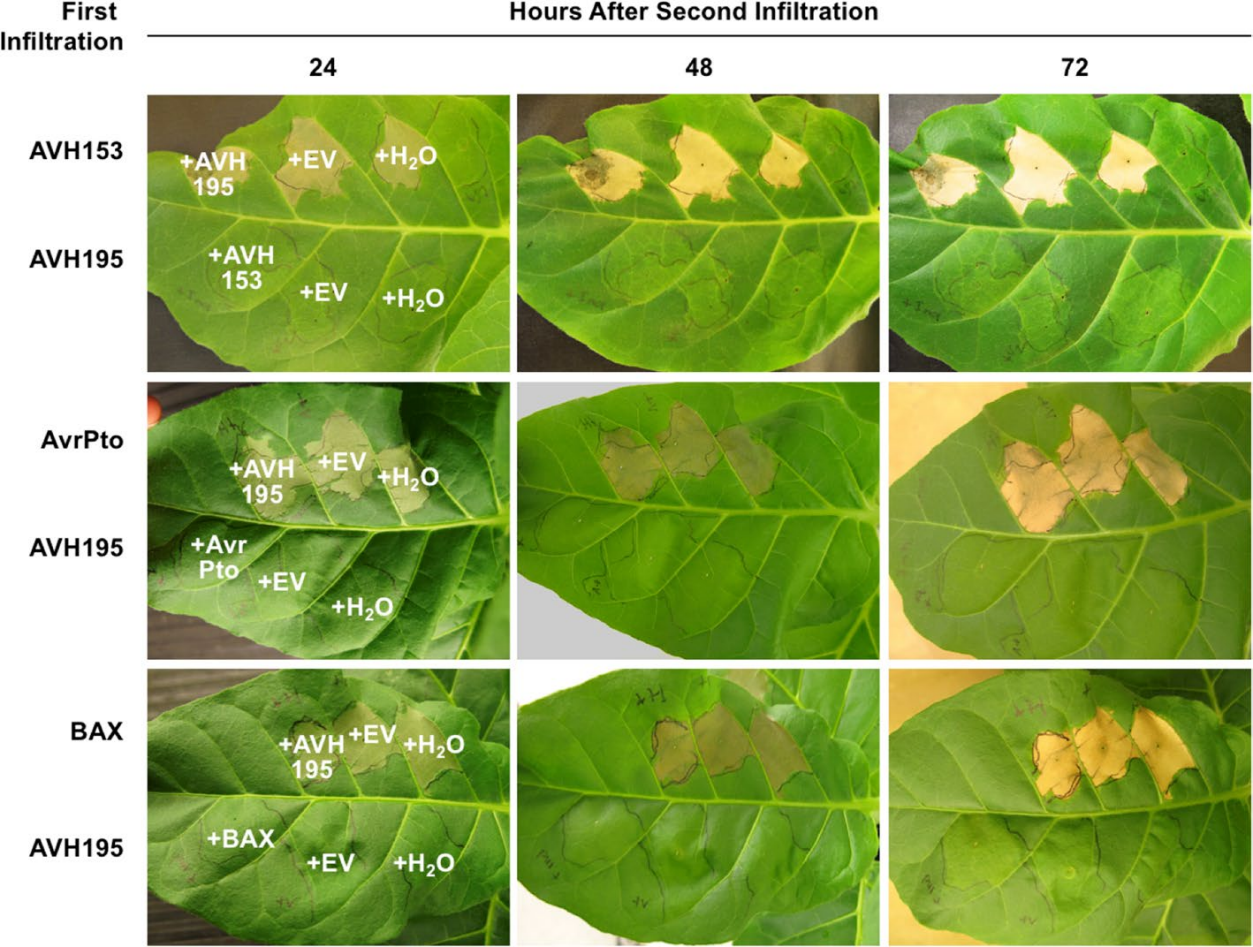
AVH195 prevents cell death*.* Agrobacterium-mediated transient expression of *AVH195* and of several cell death inducers in *Nicotiana tabacum* cv Xanthi. Agrobacterium strains containing either the AVH195 construct or one of the cell death inducers AVH153, AVRPto, and mBAX were infiltrated into leaf areas (first infiltration) 24 h before a second infiltration was performed. AVH195 prevents cell death caused by all inducers, but only when it is inoculated 24 h before infiltrations with the cell death inducers, i.e., when it is present in the leaves before cell death is initiated. When the cell death pathways are induced 24 h before AVH195 is expressed, the effector is not able to restrict the processes. Symptoms were recorded 24, 48, and 72 h after the second inoculation. Each row shows the same leaves that remained attached to the plants

The expression of AVH195 during infection suggests that the effector contributes to biotrophy. This could be achieved either by creating a favorable environment for biotrophic growth (e.g. during haustorium formation) or by preventing the premature onset of cell death and transition to necrotrophy. To investigate whether AVH195 is able to influence cell death, we set up a complex assay in which the cell death-inducing effector protein AvrPto from *Pseudomonas syringae* [22] and the proapoptotic Bcl-2-associated X protein (BAX) [23] were transiently expressed together with AVH195 in *Nicotiana* at a time interval of 24 h (Fig. 2). We also included the cell death-initiating effector AVH153 in the analysis, a *P. parasitica* ortholog of the *P. infestans* effector AVR3b [24] (Additional file 2: Fig. S1). AVH153 expresses its cell death-inducing activity in tobacco, but not in *N. benthamiana*, similar to what has been observed for AVR3b [25]. We therefore used *N. tabacum* instead of *N. benthamiana* for transient expression in our cell death studies. When AVH153, AvrPto, or BAX were expressed in leaf tissue 24 h before the addition of AVH195, cell death developed in all cases and was not affected by AVH195 (Fig. 2A, upper half of the leaves). Similar observations were made when Agrobacteria expressing AVH195 and the cell death elicitors were simultaneously infiltrated into the leaf areas (Additional file 2: Fig. S2). In contrast, when AVH195 was expressed first and the death elicitors were added 24 h later, the latter were unable to induce cell death in the transient expression region (Fig. 2A, lower half of leaves). The observations indicate that AVH195 is able to prevent the initiation of cell death independently of its elicitor, but that the effector is not able to inhibit or arrest cell death once it has been initiated. These findings suggest that AVH195 interferes at a central cell death initiation node that is targeted by AVH153, AvrPto, and BAX. They also suggest that AVH195 contributes to the biotrophic growth of *P. parasitica* in plant tissue by preventing or delaying the transition to necrotrophy.

### AVH195 co-localizes with ATG8 at endomembranes

**Fig. 3:**
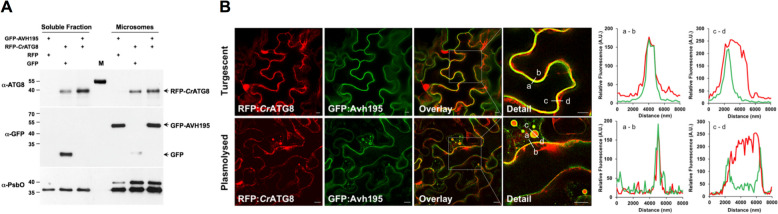
AVH195 co-localizes with ATG8 at endomembranes*.* **A** Immunoblotting of soluble and microsomal fractions prepared from *N. bentamiana* leaves transiently co-expressing GFP-tagged AVH195 with free RFP, RFP-tagged *Cr*ATG8 with free GFP, and GFP-tagged AVH195 with RFP-tagged *Cr*ATG8. Soluble and membrane-associated proteins were revealed with antisera directed against ATG8 and GFP. An antibody recognizing photosystem II PsbO, present in soluble and membrane-associated forms, was used as a loading control. Note that the ATG8 antiserum recognizes the 55-kDa pre-stained protein of the PageRuler Protein Ladder (M). **B** Optical sections of *N. benthamiana* epidermal cells transiently co-expressing AVH195 and ATG8 from *C. reinhardtii*, as analyzed by confocal laser scanning microscopy. The analyzed cells were either turgescent or plasmolysed to reveal N-terminal RFP-tagged *Cr*ATG8 (left column), N-terminal GFP-tagged AVH195 (second column from left), or the co-localization of both as yellow-orange color in overlay channel micrographs (third column from left). Squares denote details shown magnified in the right column. Relative fluorescence intensity plots of the GFP and RFP signals shown in the two columns on the right were acquired in the corresponding detailed overlay panels along the lines from a to b and from c to d. Bars represent 10 µm

Autophagy is one of the central nodes for promoting cell death in plant tissues [26, 27]. The occurrence of multiple AIMs in the AVH195 effector domain suggests that AVH195 may impair autophagy by interacting with ATG8. *ATG8* occurs as a multigene family in plants [28]. The Arabidopsis genome has nine *ATG8* genes [29], which show differential expression in different tissues and respond differently to environmental stimuli [7]. To circumvent selectivity/specificity for a particular ATG8 isoform and avoid the question of which of the 9 Arabidopsis proteins we should choose, we used the single-copy gene encoding ATG8 in the green alga *C. reinhardtii* for transient expression studies in *N. benthamiana* [30]. We generated gene constructs for N-terminal green fluorescent protein (GFP)-tagged AVH195 without the signal peptide and N-terminal red fluorescent protein (RFP)-tagged ATG8 from *C. reinhardtii* (*Cr*ATG8)*.* Subsequently, soluble and microsomal proteins were prepared from *N. benthamiana* leaves expressing the constructs and analyzed by Western blotting. An anti-ATG8 antiserum detected RFP:CrATG8 in almost equal amounts in both the soluble and microsomal fractions (Fig. 3A, top blot), consistent with the two states of the protein during autophagy [31]. In contrast, GFP-tagged AVH195 was only detected in the microsomal fraction (Fig. 3A, middle blot).

We then investigated whether AVH195 and ATG8 co-localize in the same subcellular compartments of plant cells. The GFP:AVH195 construct was transiently co-expressed with RFP:CrATG8, and leaf sections expressing the proteins were analyzed by confocal laser scanning microscopy (CLSM). Confocal imaging showed that the fluorescence of GFP:AVH195 was homogeneously distributed at the edges of the fully turgescent epidermal cells. RFP fluorescence associated with CrATG8 was also found at these edges, and co-localization of the two proteins was evident in overlay images and corresponding relative fluorescence intensity plots (Fig. 3B, first row). RFP fluorescence of CrATG8 was additionally observed in the cytoplasm and sometimes in the nucleus (Fig. 3B, first row). The localization pattern of both proteins became more evident in plasmolyzed epidermal cells (Fig. 3B, second row). Here, GFP:AVH195 and RFP:CrATG8 were co-localized at the main endomembrane structure and at membranes surrounding vesicles, which in turn contained free RFP:CrATG8 (Fig. 3B, second row). These finding suggests an interaction between AVH195 and membrane-associated ATG8 at the endomembrane system of the plant cell.

### AVH195 associates with ATG8 at the vacuolar membrane

**Fig. 4:**
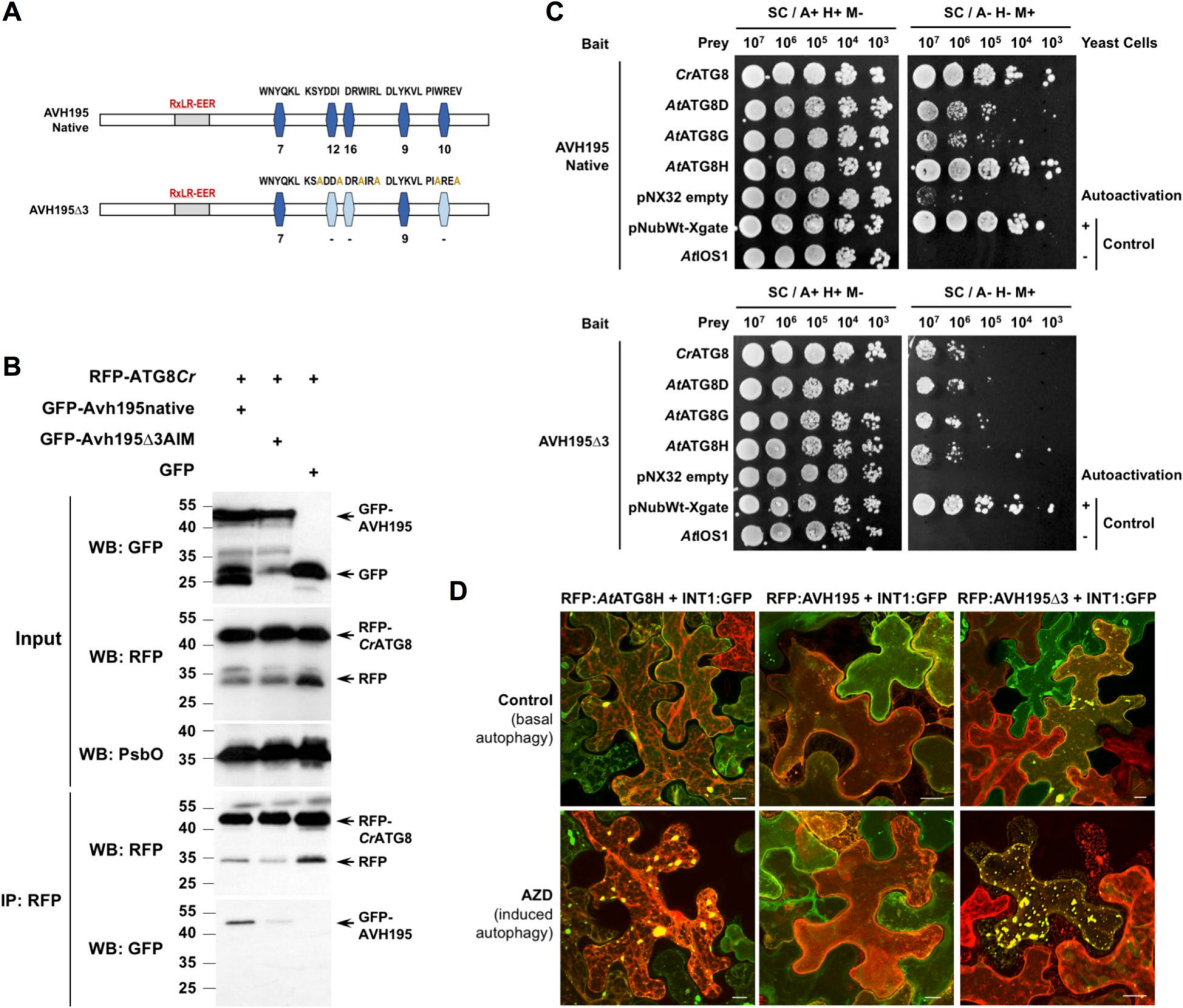
AVH195 interacts with ATG8 at the tonoplast. **A** Graphic representation of native AVH195 and the AIM mutant variant AVH195∆3. Blue rectangles with truncated edges represent predicted AIMs. Light-blue rectangles represent mutated AIMs. Corresponding sequences are indicated above the rectangles, and iLIR PSSMs below them. **B** Co-immunoprecipitation (Co-IP) of RFP-tagged *Cr*ATG8 and GFP-tagged AVH195 variants from microsomal fractions of *N. benthamiana* leaves transiently expressing the proteins. Protein extracts (input) were immunoprecipitated with anti-RFP beads, and immunodetected using anti-RFP and anti-GFP antibodies. The anti-PsbO antibody was used as the input loading control. **C** The mbSUS yeast two-hybrid system for proteins interacting on membranes was employed with AVH195 variants and ATG8 isoforms from *C. reinhardtii* and *A. thaliana*. Co-expression of the native AVH195 bait with ATG8 preys (upper images) allows yeast cells to grow on selective medium in the absence of adenine (A) and histidine (H), and in the presence of methionine (M). The AVH195∆3 bait variant decreases the capacity of yeast cells to grow on selective medium (lower images). The negative prey control *AtIOS1* encodes the membrane-anchored exodomain of the Arabidopsis receptor protein, IOS1. **D** Maximum projection images from merged optical section stacks of plasmolyzed epidermal cells of *N. benthamiana* transiently co-expressing RFP:*At*ATG8H, RFP:AVH195, or RFP:AVH195∆3 with the tonoplast-located INT1:GFP protein fusion from Arabidopsis. Fourty-two hours after Agrobacterium-mediated transformation, the inoculated leaf areas were infiltrated with either the control solution (0.01% DMSO; top row), or the autophagy inducer AZD8055 (2 µM in 0.01% DMSO; bottom row), before analyzing RFP and GFP fluorescence by confocal laser scanning microscopy. Merged signals from N-terminal RFP-tagged proteins and C-terminal GFP-tagged INT1 reveal co-localization as yellow-orange color. Bars represent 10 µm. Split images of individual focal sections and relative fluorescence intensity plots of GFP and RFP signals are shown in Additional file 2: Fig. S4

To determine whether AVH195 and ATG8 can interact and whether the AIMs influence the potential interaction, we generated a GFP:AVH195∆3 variant by site-directed mutagenesis in which hydrophobic amino acids of the three AIMs with an iLIR PSSM of ≥ 10 were changed to alanine (Fig. 4A). We then performed co-immunoprecipitation experiments with membrane-associated proteins from leaves of *N. benthamiana* transiently expressing GFP-tagged AVH195 and AVH195∆3 with RFP-tagged *Cr*ATG8. Solubilized microsomal proteins were subjected to RFP trapping. Analysis of the proteins by Western blotting showed that the trapped RFP:*Cr*ATG8 was associated with GFP:AVH195 (Fig. 4B). This association was impaired when co-expression of RFP:*Cr*ATG8 was performed with the AIM mutant variant GFP:AVH195∆3 instead of the tagged native protein (Fig. 4B). These findings indicate an AIM-dependent association between AVH195 and ATG8 at the membranes.

To extend the analysis, we used the mating-based split-ubiquitin yeast two-hybrid system (mbSUS), which has been developed to detect the interactions between membrane-anchored proteins and their partners [32]. Yeast transformants expressing the native AVH195 bait with the *Cr*ATG8 prey grew readily on selective medium, indicating an interaction between the two proteins (Fig. 4C, upper spotting). We then tested three members from the ATG8 family of Arabidopsis, namely *At*ATG8D, *At*ATG8G, and *At*ATG8H, which are representatives of the three established phylogenetic clades that constitute this multigene family [29] (Additional file 2: Fig. S3). Yeast cells expressing the native AVH195 bait and the *At*ATG8 preys were able to develop on selective medium, although vigor of growth was strongest with *At*ATG8H as the prey and weaker with *At*ATG8D and *At*ATG8G (Fig. 4C, upper spotting). Yeast cells expressing the AVH195∆3 AIM mutant as bait and the different ATG8 as prey were severely compromised in their growth on selective medium, indicating that the interaction of AVH195 with the different ATG8 isoforms at the membrane is mediated by the AIMs of the effector (Fig. 4C, lower spotting).

Our aim was then to determine at which subcellular membrane structures the interaction between AVH195 and ATG8 takes place. The previous observations of co-localization between GFP:AVH195 and RFP:*Cr*ATG8 suggested that the association may occur at vesicular and vacuolar membranes (Fig. 3B). This would be consistent with a role for ATG8 in vacuolar fusion and cargo delivery into the cell sap [31]. We therefore employed a gene construct encoding the GFP-tagged inositol transporter 1 (INT1) from Arabidopsis, which is specifically localized to the tonoplast [33, 34]. We changed the fluorescent tag of AVH195 from GFP to RFP, transiently expressed INT1:GFP with either RFP:*At*ATG8H, RFP:AVH195, or the RFP:AVH195∆3 variant in *N. benthamiana*, and analyzed the subcellular localization of the proteins in plasmolyzed cells using CLSM. Red fluorescence of *At*ATG8H (Fig. 4D, upper left image; Additional file 2: Fig. S4, first row), AVH195 (Fig. 4D, upper middle image; Additional file 2: Fig. S4, second row), and AVH∆3 co-localized with the green fluorescence of INT1 at the membrane. It should be noted that RFP:AtATG8H, similar to RFP:AVH195∆3, occasionally aggregated with INT1:GFP in large speckles, whereas the native variant RFP:AVH195 did not, but rather distributed evenly with INT1:GFP along the membrane (Additional file 2: Fig. S4, upper three rows). The appearance of these speckled ATG8-INT1 aggregates increased in number and intensity when the autophagy inducer AZD8055 [35] was applied to leaf areas that have previously been transformed with the gene constructs (Fig. 4C, lower left image; Additional file 2: Fig. S4, fourth row). The enhanced aggregation of INT1:GFP was not observed in AZD-8055-treated leaf areas co-expressing the native RFP:AVH195 variant (Fig. 4C, lower middle image; Additional file 2: Fig. S4, fifth row), but appeared in AZD-8055-treated leaf areas, when the RFP:AVH195∆3 variant instead of the native one was co-expressed with INT1. These experiments show that AVH195 and ATG8 co-localize with INT1 at the vacuolar membrane. The tonoplast thus appears to be the subcellular structure at which the interaction between AVH195 and ATG8 takes place. The experiments also indicate that stimulation of autophagy leads to the formation of aggregates at the tonoplast that accumulate ATG8 and INT1. This aggregate formation appears to be largely reduced in the presence of native AVH195, whereas the AIM mutant variant AVH195∆3 appears unable to prevent it. Our observations taken together suggest a role for the effector in attenuating the autophagy cycle.

### AVH195 delays vacuole fusion and autophagic degradation in the green alga C. reinhardtii

We showed that AVH195 can interact with ATG8 from *C. reinhardtii.* The green alga has become a model organism for studies on a variety of fundamental cellular processes in *Viridiplantae*, including development, reproduction, chloroplast biology, and photosynthesis [36]. We therefore investigated possible perturbations caused by the interaction between AVH195 and ATG8 on cellular autophagic processes in this reduced complexity model provided by the unicellular photosynthetic alga. To estimate whether AVH195 interferes with the core autophagic process, we generated *C. reinhardtii* transformants expressing the effector gene. The nuclear genome of the alga has a GC content of ~ 65% [37]. We therefore designed a synthetic *AVH195* coding region (without the signal peptide) with a codon usage adjusted to the alga and introduced it into the cell line *dw15.1*, which is devoid of a cell wall. Transformants were selected for the presence and expression of the transgene. We retained three independent lines, N15, N26, and C40 for further analysis. Wild-type (Wt) and transgenic cell lines were then synchronized by alternating light and dark periods over 24 h. To assess effects of *AVH195* expression on *Chlamydomonas* physiology, we analyzed several vital parameters by spectral flow cytometry over the 24-h circadian cycle (Additional file 2: Fig. S5). Cell size was measured by forward light scattering (FSC), and the complexity of cellular structure by side light scattering (SSC). Autofluorescence of the cells reflected potential changes in the chlorophyll content of cultures and the addition of 4′,6-diamidino-2-phenylindole (DAPI) to the cells allowed evaluating cell death. Finally, the cell proliferation rate was assessed by measuring the dilution of the fluorescence intensity of 5-,6-carboxyfluorescein succinimidyl ester (CFSE) upon cell division [38]. The different parameters were generally closely linked to the circadian cycle. Cell proliferation predominantly occurred during the night, but stagnated under light, whereas all other parameters (including DAPI stain) reached a peak after 12 h of light and decreased during the night until reaching a level similar to that observed at the beginning of the analysis. Wild-type (Wt) and transformant cultures increased cell number with similar kinetics and to similar magnitudes over the light period and decreased likewise (Additional file 2: Fig. S5). Only slight differences between cell lines were observed, which likely reflect individual variations rather than a global trend. We thus concluded that expression of *AVH195* had no incisive effect on the physiology of *Chlamydomonas* cells.

**Fig. 5:**
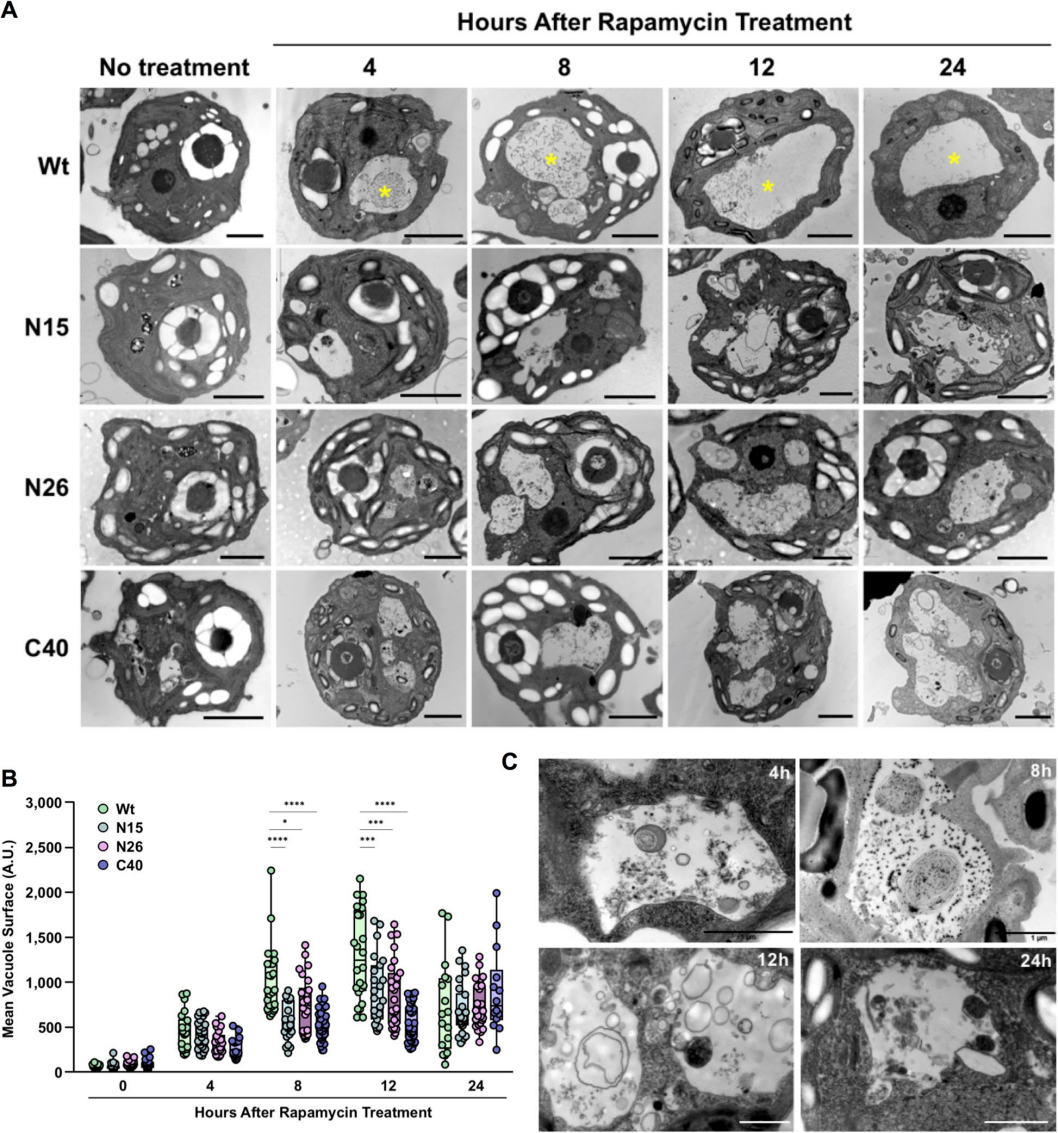
Transmission electron microscopy of the subcellular phenotypes of *AVH195*-expressing *Chlamydomonas* cells. **A** Under non-induced conditions, wild-type (Wt) cells (upper lane) contain multiple empty vacuoles that fuse into a dominant large vacuole (yellow asterisks), 4 h after addition of rapamycin. Cells expressing AVH195 display a delay in central vacuole fusion, when compared to the Wt. No obvious other modifications in the subcellular organization are related to effector expression. The bars represent 2 µm. **B** Rapamycin-induced vacuole swelling is impaired in transformants expressing *AVH195*. Here, a dominant vacuole is replaced by smaller vacuoles. For each line and time point of treatment, micrographs from 4–5 independent TEM sections representing 10–12 cells each were analyzed for the presence of vacuoles. The surface of the biggest vacuole in each cell was then determined using the ImageJ software. Shown are Box Whisker plots with all points from *n* = 14–30 vacuoles per line and time point of treatment. Significance groups indicated by stars above the boxplots were determined by one-way ANOVA with Tukey’s multiple comparisons test (*, *p* < 0.05; ***, *p* < 0.001; ****, *p* < 0.0001). A.U. = Arbitrary Units. **C** Higher resolution views of *AVH195*-expressing cells from line N26 presenting lysosome-like vacuoles containing electron dense material. This material started to accumulate in vacuoles 4 h after rapamycin treatment. The non-degraded cargo persists without elimination over 24 h of autophagy induction. Bars represent 1 µm. For representative views at lower magnification, see Additional file 2: Fig. S6

We stimulated autophagy by supplementing the algal cultures with 0.5 µM rapamycin, a concentration that has no effect on the vital parameters of *Chlamydomonas* [39]. The subcellular organization of untreated and rapamycin-treated algae was then analyzed by transmission electron microscopy (TEM). Upon rapamycin treatment, empty vacuoles fused to form a predominant large vacuole in Wt cells as soon as 4 h after onset of treatment (Fig. 5A; Additional file 2: Fig. S6, upper lanes). Conversely, cells overexpressing *AVH195* displayed a marked accumulation of nondigested material in lysosome-like structures, and a delay in central vacuole fusion, when compared to the Wt (Fig. 5A; Additional file 2: Fig. S6). To determine the dynamics of vesicle fusion and the formation of a central vacuole in a quantitative manner, we measured the surface of the biggest vacuole in individual cells on 4 to 5 TEM micrographs that represent about 10 cells each. The surface of vacuoles in Wt cells increased constantly to maximum sizes at 12 h after rapamycin treatment, before decreasing at 24 h (Fig. 5B). By contrast, cells from the 3 transgenic lines appeared impaired in vacuole merging, as they maintained significantly smaller vesicles over the initial 12 h of treatment. The differences in vacuole size between the Wt and the transgenics were no longer observed after 24 h of treatment (Fig. 5B). At higher TEM resolution, the transformed cells showed an accumulation of various cytoplasmic debris and organelle-like structures upon rapamycin treatment (Fig. 5C). We suspect that these structures were autophagic bodies and their debris that accumulate in the vesicles of transformed cells. Overall, the data suggest that AVH195 interacts with ATG8 from *C. reinhardtii* to decelerate autophagic turnover in the alga, as reflected by delayed vacuole formation, the inability of small vesicles to fuse with vacuoles, and the accumulation of cell debris in vesicles.

### AVH195 promotes biotrophic oomycete development in Arabidopsis

We then aimed to analyze whether the abovementioned properties of AVH195 influence pathogenicity of the oomycete. We generated transgenic Arabidopsis lines that express AVH195 under the control of the constitutive Cauliflower Mosaic Virus (CaMV) *35S* promoter (*p35S*) and used two of them (lines OE6 and OE9) for further studies. We first analyzed them for autophagic turnover, as its slowdown was observed as a result of AVH195 activity in *Chlamydomonas*. Autophagic turnover (or flux) involves the recycling of membrane-associated ATG8. During autophagy, cytosolic ATG8 is cleaved at the C-terminus, conjugated with phosphatidylethanolamine and bound to vesicle membranes. After vesicle expansion and fusion, ATG8 is then either degraded or released from the membranes and recycled. The turnover of ATG8 at endomembranes is thus an indicator of a functioning autophagic flux.

**Fig. 6:**
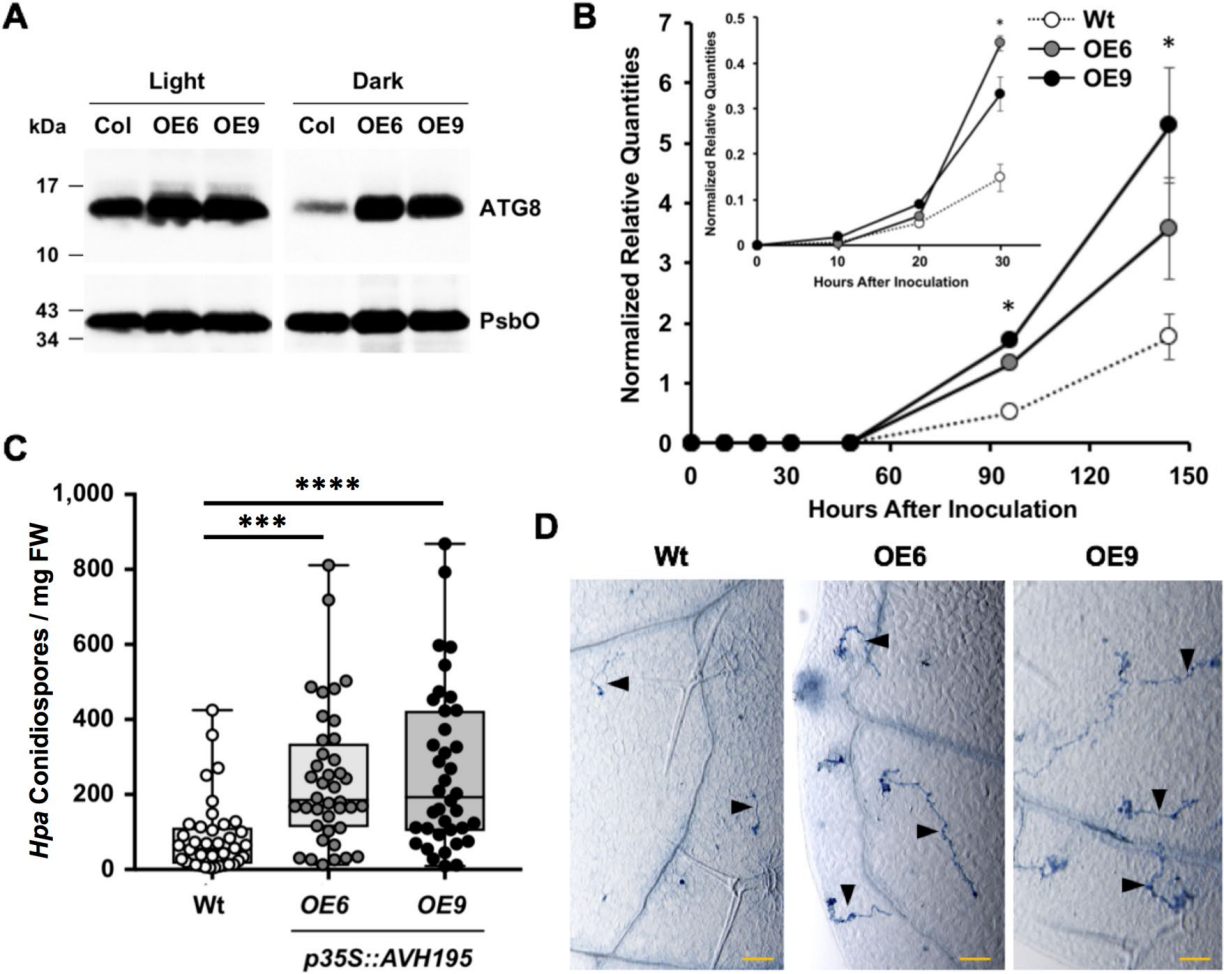
Expression of *AVH195* in transgenic *A. thaliana* attenuates ATG8 turnover and increases susceptibility to Oomycete pathogens*.* **A** Immunoblot showing accumulation of ATG8 in microsomal fractions from the wild-type (Col) and from 2 independent transgenic (*OE6* and *OE9*) lines expressing *Avh195*. Upon autophagy induction in the dark, transgenic lines maintain higher quantities of membrane-associated ATG8 than the Wt. An antibody recognizing the PsbO protein was used for loading control. **B** Biomass of *P. parasitica* develops faster and stronger in *AVH195*-expressing plants, when compared to the Wt. RNA extracted from inoculated samples were analyzed by RT-qPCR for the accumulation of transcripts from the constitutively expressed *P. parasitica* gene *WS41*. The insert shows the evolution of biomass during the first 30 h post inoculation in the same experimental onset. Shown are the means (± SD) from 3 biological replicates. Asterisks indicate significant differences with *p* ≤ 0.05, according to the nonparametric Mann–Whitney test. **C** Increased *Hyaloperonospora arabidopsidis* (*Hpa*) conidiospore production on plants from the *AVH195*-overexpressing lines, when compared to plants from the wild-type. Shown are the amounts of conidiospores per mg fresh weight (FW) from two biological replicates each consisting of 20 samples. Statistically significant differences were determined by one-way ANOVA with Tukey’s multiple comparisons test. Significance groups are represented by asterisks (***, *p* < 0.001; ****, *p* < 0.0001). **D** Invading *Hpa* hyphae (arrowheads) develop better in *AVH195-*expressing plants, when compared to the Wt. Infected leaves from the Wt and the transgenic lines were collected 24 h post inoculation and stained with trypan blue. The micrographs show representative focused sections. Bars represent 100 µM. See Additional file 2: Fig. S7 for more images

We induced autophagy in Wt and AVH195-expressing Arabidopsis plants by growing them in the dark [40]. We prepared microsomal proteins from these plants and from plants grown under normal light conditions and subjected them to immunoblot analysis for ATG8. In Wt Arabidopsis grown in the dark, we found only small amounts of ATG8 accumulating at the membranes, indicating that ATG8 turnover functions in these plants in the dark (Fig. 6A). In contrast, the transgenic lines accumulated larger amounts of membrane-associated ATG8, suggesting that turnover of the protein is reduced in plants expressing *AVH195* (Fig. 6A).

To evaluate the consequences for pathogen development, seedlings from homozygous transgenic lines and from the Wt were inoculated with zoospores of *P. parasitica*. The development of the oomycete in plant tissues was then determined by RT-qPCR using the constitutively expressed *P. parasitica* gene *WS41* as a marker for developing oomycete biomass in plant tissues [41, 42]. *Phytophthora* transcripts were clearly detectable from 20 hpi and accumulated at a significantly higher level in the two transgenic lines, when compared to the Wt (Fig. 6B). From this we concluded that plants expressing *AVH195* were significantly more susceptible to *P. parasitica* than Wt plants. Since the effector gene is predominantly expressed during the biotrophic phase of infection (Fig. 1B) and represses cell death (Fig. 2), we presumed that AVH195 particularly promotes biotrophic pathogen growth. Therefore, we inoculated plants from the transgenic lines and from the Wt with the obligate biotrophic oomycete pathogen, *Hyaloperonospora arabidopsidis* (*Hpa*). To estimate the infection success, we determined the amount of asexual conidiospores that were produced at the end of the infection cycle [43]. Sporulation rates were significantly higher in plants of the transgenic lines expressing *AVH195* than the Wt (Fig. 6C). To determine whether the increase in sporulation rates in the transgenic lines correlated with better growth of the oomycete in plant tissue, we analyzed the extent of hyphal growth in leaves at an early infection stage (24hpi) in plants from the Wt and transgenic lines. In both transgenic lines, initial hyphae developed faster than in the Wt (Fig. 6D; Additional file 2: Fig. S7). All data taken together therefore strongly suggest that AVH195 promotes biotrophic growth of oomycete pathogens in plant tissues by interacting with ATG8 of the host and delaying autophagic flux.

## Discussion

We show here that the *P. parasitica* effector AVH195 interferes with the plant autophagy machinery through interaction with the host ATG8. AVH195 belongs to the RxLR superfamily of cytoplasmic effectors, which play a major role in plant infection. All oomycete avirulence genes (*AVRs*) identified to date encode effectors of the RxLR family, and generally display important natural allelic variation (including deletions), differential gene expression, and copy number variation as an adaptive evolution to evade resistance gene-mediated plant immune responses [44]. Unlike many other *AVRs*, *AVH195* displays a particularly low rate of polymorphism among natural populations, which does not affect the predicted physico-chemical properties of the encoded proteins. We therefore conclude that AVH195 is under strong purifying selection. Although cases of host specificity have been reported, *P. parasitica* is generally a versatile *Phytophthora* species that infects hundreds of host plants [15]. The conservation of AVH195 indicates that this effector can be considered as a core virulence component of *P. parasitica* and does not contribute to host range delineation. We therefore expected that the pathogenicity target of AVH195 is similarly conserved in plants and that it contributes to an essential physiological mechanism. The observation that the effector is able to prevent the onset of cell death, which is equally activated by different AVR products and the proapoptotic BAX protein, but cannot stop cell death once it has started, suggests that AVH195 targets a central mechanism upstream of the death pathways. The observed interaction of AVH195 with ATG8s from Arabidopsis and *C. reinhardtii* points to autophagy as this central mechanism.

The expansion of ATG8 isoforms in higher plants suggests functional diversification of the protein [28]. Genes encoding ATG8 have diverged from a common ancestor into three subgroups during the speciation of plants and present differential spatiotemporal expression profiles in *A. thaliana* [29]. However, proteins from the ATG8 family are highly conserved within the green lineage and the amino acid sequences of isoforms from Arabidopsis are frequently more than 80% identical with the single ATG8 sequence from *C. reinhardtii*. ATG8 from the green alga may thus reflect an ancestral form of the protein and might represent a common denominator for ATG8 isoforms from the different plant species that are hosts for *AVH195*-expressing *P. parasitica* isolates. This assumption prompted us to use *C. reinhardtii* to analyze the role of AVH195 in recipient cells. To our knowledge, the here-presented physical interaction between AVH195 and CrATG8, as well as our data on the consequences of this interaction for the cell biology of the alga, represent the first report that an oomycete effector might be active in a non-plant, but photosynthetic organism. Trans-kingdom activity of an effector has so far only been demonstrated for a CRinkler and Necrosis (CRN) protein from the legume root pathogen *Aphanomyces euteiches.* This effector has been successfully expressed in *Xenopus laevis* embryos, where it triggers aberrant developmental modifications [45]. We did not observe aberrant phenotypes that affect vital parameters of the *AVH195*-expressing *Chlamydomonas* cells, making the alga a promising model for further effector studies. TEM analyses of rapamycin-treated *Chlamydomonas* cells revealed the presence of smaller vesicles in *AVH195*-expressing cells than in cells from the Wt, and a delay in the appearance of large vacuoles. This observation suggests perturbation of ATG8-mediated vesicle fusion in *AVH195*-expressing cells. Furthermore, these cells accumulate cellular debris similarly to what is observed in *Chlamydomonas* cells that were treated with Concanamycin A, an inhibitor of autophagic flux [46].

Upon expression in *N. benthamiana*, we found AVH195 associated with microsomal membranes in plant cells, while ATG8 from *C. reinhardtii* was present in both soluble and membrane-associated protein fractions. Live cell images showed that co-localization of RFP:CrATG8 and GFP:AVH195 occurred both at vesicular membranes and at a membrane containing the tonoplast-specific INT1 protein [33, 34]. The labeled vesicles were additionally charged with free ATG8 and appeared to release this charge into the vacuole after fusion (Fig. 3B, bottom right). A notable observation was that ATG8 accumulated together with INT1 in speckled aggregates at the vacuolar membrane after stimulation of autophagy. The AIM mutant AVH195∆3 also located in such aggregates together with INT1, whereas native AVH195 did not, but rather distributed evenly with INT1 along the tonoplast (Fig. 4D, Additional file 2: Fig. S4). The speckled (or punctate) accumulation of ATG8 has previously been described as an indicator of autophagosome induction during stimulation of autophagy by the *P. infestans* effector PexRD54 [47]. Surprisingly, the C-terminal 32-amino acid peptide of the same effector (AIMp) represses autophagy, and this repression leads to a strong reduction in the number of ATG8 puncta [47]. The appearance of speckles/puncta therefore seems to be indicative for an induction of autophagy, while their disappearance rather indicates its repression. Applied to our observations, AVH195 appears to repress autophagy, while the AIM mutant AVH195∆3 has lost this activity. Taken together, AVH195 seems to interact and interfere with ATG8 at the endomembrane system of the cell. The local accumulation of ATG8 together with the tonoplast transporter INT1 seems to indicate a high autophagic activity, which is smoothed by AVH195 and reflected in a homogeneous distribution of INT1 along the vacuolar membrane. We intended to use the INT1:GFP protein as a confirmed marker protein for the tonoplast in CLSM analyses only. We admit that we were surprised when we observed the focal accumulation of INT1 with ATG8 and the AVH195∆3 mutant variant at the vacuolar membrane at the onset of autophagy (and its avoidance in the presence of native AVH195). This observation could indicate that the stimulation of autophagy leads to the formation of membrane protein aggregates, of which INT1 is a rather passive part, but not the trigger. However, it might also indicate a specific role of INT1 in autophagy. A direct link between INT1 and autophagy has not yet been reported, but the transporter is required for the recycling of *myo*-inositol in the vacuole and for its release into the cytoplasm [34]. *Myo*-inositol is a building block for intracellular signaling molecules that control a variety of cellular functions [48]. A role of inositol derivatives in the regulation of autophagy has been shown in mammalian cells, where a decrease in intracellular inositol trisphosphate levels stimulates autophagy, while their increase inhibits it [49]. Whether our studies reveal a passive or rather active role for INT1 in the AVH195-mediated regulation of autophagy remains to be investigated in future studies.

We found that Arabidopsis cells expressing AVH195 accumulate higher amounts of ATG8 in the membranes than Wt cells under autophagy-inducing conditions. Sustained accumulation of ATG8 on membranes seems be due to impaired completion of autophagy and ATG8 turnover, a defect in recycling, and an attenuation of autophagic flux [50]. All here-presented observations taken together converge to our interpretation that AVH195 interacts with ATG8 at membranes of autophagosome-like vesicles and vacuoles to delay autophagic flux.

The role of autophagy for the outcome of plant–microbe interactions is particularly complex and dependent on the nature of the pathogen [51, 52]. Through pro-death and pro-survival activities, it may contribute to either the establishment or the restriction of cell death at pathogen infection sites [10, 12]. *P. parasitica* is a hemi-biotrophic pathogen, which maintains host cells alive as a biotroph, before switching into a necrotrophic lifestyle. Upon infection of Arabidopsis, the phase switch between biotrophy and necrotrophy occurs 15 to 30 h after inoculation [20]. *AVH195* transcripts accumulate almost exclusively during the short biotrophic phase, indicating that the effector either promotes biotrophy or prepares the switch to necrotrophy and host cell death. However, AVH195 represses the initiation of cell death and the obligate biotrophic oomycete pathogen *Hpa* develops much better in *AVH195*-expressing Arabidopsis than in the Wt. We therefore suggest that AVH195 targets autophagy to delay the pro-death function of this mechanism, thus favoring biotrophic growth and establishment of *P. parasitica* in plant tissues.

Various pathogens have evolved sophisticated mechanisms to either activate or suppress autophagy in host cells to promote infection, depending on their lifestyle in the host [53]. The expansion of several *ATG* genes and the complexity of effector repertoires of pathogens may provide myriads of possible interactions involving the various members of the plant autophagy machinery. A systematic analysis identified a network of 88 potential interactions between 184 effectors from different plant pathogens with 25 Arabidopsis autophagy (ATG) proteins [9]. This analysis revealed that ATG8 in particular is a central hub for pathogen effectors [11]. Some effectors, like the *Pseudomonas syringae* effector HrpZ1, can interact with several members of the ATG8 family and promote autophagy, while HopF3, which is produced by the same pathogen, suppresses autophagy although sharing with HrpZ1 six common ATG8 isoforms for interaction [11]. The only ATG8-directed effector from an oomycete plant pathogen described to date and for which functional studies have been performed is the *P. infestans* RxLR protein PexRD54. This effector was shown to interact with ATG8CL from potato in a rather isoform-specific manner. The PexRD54 interaction with ATG8CL activates autophagy without disturbing autophagic flux [13]. The analysis of AVH195 presented here thus extends the identification of oomycete effectors that target plant autophagy for successful infection. Unlike PexRD54, which displays a discriminant, high affinity for potato ATG8Cl, AVH195 interacts with ATG8 isoforms from the 3 clades of proteins in Arabidopsis, and with ATG8 from *C. reinhardtii*, which may be considered as an ancestral form of the protein. In contrast to the autophagy-activating activity of PexRD54, AVH195 impairs autophagy, apparently by delaying autophagic flux. Thus, AVH195 and PexRD54 appear to have different specificities and exert distinct functions while targeting the same mechanism in host cells. An ortholog of *PexRD54* exists in the *P. parasitica* genome (PPTG_03663, XP_008894598) and *P. infestans* possesses with PITG_04099 an ortholog of AVH195. Our report therefore suggests that *Phytophthora* species produce different effectors that target host ATG8 to either attenuate or enhance host autophagy and thus adapt their environment during the course of infection. Further studies should then show how pathogens express and use these different effectors to optimize their lifestyle in the host plant.

## Conclusions

*Phytophthora parasitica* produces AVH195 during biotrophic plant invasion. The effector associates with host ATG8 at the vacuolar membrane and functions in plants and the green alga *C. reinhardtii* to attenuate autophagic flux. It suppresses cell death and, when expressed in Arabidopsis, promotes the biotrophic infection by oomycetes. The data suggest that AVH195 belongs to a first wave of effectors that favor biotrophy, allowing the pathogen to suppress rejection and to settle stably in host tissues during the initial phase of infection.

## Methods

### Plant material and oomycete cultures

Arabidopsis (Col-0) plants were grown in growth chambers at 21 °C as described [54]. *Nicotiana benthamiana* and *N. tabacum* were cultivated as described [55]. *P. parasitica* was maintained in the *Phytophthora* collection at INRAE, Sophia Antipolis, France. The conditions for *P. parasitica* in vitro growth and zoospore production, and for cultivation of *Hyaloperonospora arabidopsidis* (*Hpa*) isolate Noco2 were previously described [43, 56].

### Chlamydomonas reinhardtii culture and transformation

The *C. reinhardtii* strain *dw15.1* (nit1-305 cw15; mt +), a cell wall-less derivative of strain *cw15*, was used for all studies. Seed cells were cultivated in 250-ml flasks with 100 ml Tris Acetate Phosphate (TAP) medium [57] with a 12 h photoperiod at 25 °C. Rapamycin (1 mg/ml stock in 90% ethanol-10% Tween 20) was added to cultures at a final concentration of 0.5 µM. The synthetic *AVH195* gene was designed using the average *C. reinhardtii* codon usage implemented in the Codon Usage Database (http://www.kazusa.or.jp/codon) and a manual optimization. The gene was synthesized by GeneArt technologies and cloned into the *Kpn*I and *Not*I restriction sites of the pChlamy_3 vector (Thermo Fisher Scientific). Nuclear transformation was performed as previously described [58]. Electroporation was performed by applying an electric pulse of 0.7 kV at a capacitance of 50 µF (Gene Pulser, Bio-Rad), using 400 ng of *ScaI*-linearized plasmids purified by agarose gel electrophoresis. Transgenic strains were selected directly on TAP/agar plates containing hygromycin B (10 mg/L), and the plates were incubated under continuous fluorescent light (20 µmol m^−2^ s^−1^) at 25 °C before being transferred to the above-described culture conditions.

### Sequence analysis, vector construction, and site-directed mutagenesis

For sequencing, total genomic DNA was extracted as described [59] from 4-day-old mycelia of the various *P. parasitica* isolates (Additional file 1: Table S1). *AVH195* analogs were amplified with specific oligonucleotides designed with the Primer3 Plus online tool (www.primer3plus.com). Sequencing experiments were conducted at least twice on each DNA strand. Pairwise alignments were performed using Lalign (https://embnet.vital-it.ch). The intron-free coding sequence of *AVH195* from strain PPINRA-310 without the signal peptide was amplified from genomic DNA with Gateway-compatible primers. Three primer pairs were designed to alter selected AIMs of *AVH195* by site-directed mutagenesis with the QuickChange II kit (Agilent) according to the manufacturer’s recommendations. *AtATG8* coding sequences were amplified with Gateway-compatible primers from the plasmids G22544, U17226, and G82070 (ABRC, Ohio State University), which contain the Arabidopsis genes encoding ATG8D, G, and H, respectively. The *CrATG8* coding sequence was amplified with Gateway-compatible primers from Chlamydomonas *dw15.1*-derived cDNA. To enable expression of the native protein, as well as N- and C-terminal protein fusions, two sets of amplicons were generated that contained and did not contain the stop codon. All amplicons were recombined in the entry vector pDONR207 (Invitrogen) for further swaps to destination vectors. Destination vectors were pK7FWG2, pK7WGF2, and pK7WGR2 (Plant Systems Biology, VIB, Gent) for transient and stable expression in *Nicotiana* and Arabidopsis, respectively, and pMetYC-DEST and pNX32-DEST for Y2H analysis using the mbSUS system [32]. Entry-to-destination swaps were performed via Gateway LR clonase reactions (Thermo Fisher Scientific), according to the instructions of the supplier. All primers used in this study are compiled in Additional file 1: Table S2.

### Stable and transient overexpression in planta

Arabidopsis plants were transformed using the dipping method [60] and selected on MS medium plates (1% agar) containing 50 µg/ml kanamycin. Transformed plants were transferred to soil, and seeds were collected. Ten independent primary transformants (T1) harboring the constructs were verified by PCR, and homozygous T3 plants were obtained for further analysis. For transient expression analysis, *A. tumefaciens* strain GV3101 transformed with the various constructs was grown in LB medium supplemented with 50 µg/ml rifampicin, 20 µg/ml gentamicin, and 100 µg/ml spectinomycin until OD_600_ reached 1.0. Cells were pelleted, resuspended in infiltration buffer (10 mM MgCl_2_, 10 mM 2-[N-morpholino] ethanesulfonic acid [MES], pH 5.6, 150 µM acetosyringone), and adjusted to an appropriate optical density, then left for 3 h at room temperature in the dark before infiltration. The abaxial side of leaves was infiltrated using a syringe without needle. For confocal imaging, protein extraction, and subcellular fractionation, infiltrated areas were collected 72 h after infiltration and mounted either in water or in 0.8 M sorbitol prior to analysis. For the induction of autophagy, AZD-8055 (CliniSciences, Nanterre, France) was infiltrated at 2 µM in 0.01% DMSO into previously agroinfiltrated areas, 42 h after applying the Agrobacteria. Samples were then analyzed by CLSM, 6 h after AZD-8055 treatment.

### Infection assays with oomycetes

For inoculation with *P. parasitica*, *Arabidopsis* seeds were surface-sterilized in 20% NaClO/80% EtOH, rinsed 3 times with 100% ethanol, and sown on MS medium containing 2% sucrose and 2% agar. Seeds were cold-stratified for 2 days and then maintained at 21 °C under short-day conditions (8 h light / 16 h dark). After 6 days, plantlets were transferred to 96 well plates containing per well 30 µl of the same medium, which was overlaid with 25 µl of liquid 0.5 × MS containing 1% sucrose. Plants were grown for another 8 days under these conditions, before inoculation with 1000 zoospores per well. All experiments were performed in duplicates, and plantlets that were collected at different time points after inoculation were immediately frozen in liquid nitrogen before analysis by RT-qPCR. Infection assays with *Hpa* isolate Noco2 were performed and sporulation levels were determined as described [43]. For microscopic analysis of the *Hpa* infection success after 24 h, leaves were collected, stained with Trypan blue as described [43] and analyzed with a Zeiss Axio Zoom.v16 microscope.

### RNA extraction and gene expression analysis

RNA extractions were performed as described [17]. After DNAse I treatment (Ambion), 1 µg of RNA was reverse-transcribed using the IScript cDNA synthesis kit (Bio-Rad). qPCR experiments were performed in an AriaMX (Agilent) thermocycler with 5 µl of a 1:20 cDNA dilution, and 7.5 µl of Brilliant III Ultra-Fast SYBR Green QPCR Master Mix (Agilent), according to the manufacturer’s instructions. Primer pairs for qPCR were designed using Primer3 Plus. Relative expression of the target genes was normalized for Arabidopsis with transcripts from the constitutive genes At5g62050 and At5g11770 [43], for *P. parasitica* with transcripts from *WS41* and *UBC* [55]. Values were displayed as the normalized relative quantities that were determined by the modified ΔCt method employed by the qBase 1.3.5 software.

### Protein methods

Leaf sections from Agrobacterium-infiltrated *N. benthamiana* were ground in a mortar under liquid nitrogen and suspended in extraction buffer (10 mM Tris–HCl buffer at pH 7.5 containing 100 mM NaCl, 0.5 mM EDTA, and 1% Sigma-Aldrich P9599 protease inhibitor cocktail). Samples were centrifuged at low speed (1500 × *g*) for 10 min at 4 °C to pellet tissue debris. Supernatants were collected, filtered through 70-µm cell strainers, and centrifuged at 100,000 × *g* for 90 min at 4 °C. Supernatants containing soluble proteins were separated from the microsomal pellets, which were washed once with extraction buffer and resuspended with a Potter homogenizer in 0.5 ml extraction buffer containing 0.2% Igepal CA-630. Protein contents were determined with the Bradford Ultra dye (Expedeon, Cambridge, UK). For co-immunoprecipitation, 150 µg of membrane protein in 500 µl buffer was incubated with 15 µl equilibrated RFP magnetic bead slurry (Chromotek rfm-20) for 1 h at 4 °C on a rotary wheel. Beads were trapped with a magnetic particle concentrator and successively washed with washing buffer (10 mM, pH 7.5, 100 mM NaCl), including (initial washes) or not including (final washes) 0.04% Igepal CA-630. Proteins were eluted from the beads in 50 mM Tris pH 6.8, 50 mM DTT, 1% SDS, 1 mM EDTA, 0.005% bromophenol blue, and 10% glycerol, and heated for 5 min to 95 °C, prior to SDS-PAGE and Western blotting. For protein extraction from *C. reinhardtii*, 10 ml of culture treated with either DMSO as the control or rapamycin at 0.5 µM for 8 h were collected by centrifugation at 4000 × *g* for 5 min and frozen in liquid nitrogen. Cell pellets were resuspended in 200 µl of extraction buffer (50 mM sodium phosphate buffer at pH 5.8 containing 10 mM KCl, 1 mM EDTA, and 1% protease inhibitor cocktail). Cells were lysed with 3 cycles of freeze-thawing between liquid nitrogen and 37 °C, transferred to 1.5 ml reaction tubes, and grown with a tube-adapted pistil. The homogenates were centrifuged at 1000 × *g* for 8 min at 4 °C, and the recovered supernatants centrifuged at 120,000 × *g* for 1 h at 4 °C. The supernatants containing soluble proteins were transferred to fresh reaction tubes. Pellets were washed with extraction buffer and resuspended in 100 µl extraction buffer containing 1% SDS. For all experiments, proteins were separated by SDS-PAGE (10% acrylamide), transferred to PVDF membranes with the Bio-Rad Trans-Blot Turbo system, and detected with primary antibodies against ATG8 (Agrisera AS14 2769), GFP (Roche 11814460001), RFP (Genscript A00682-40), and PsbO (Agrisera AS06 142–33).

### Yeast two-hybrid analysis

The mating-based split-ubiquitin system (mbSUS) was used and employed as described [32]. Sequences encoding native or mutant AVH195 variants were integrated as baits into pMetYC-DEST and transferred into the haploid Mat_a_ yeast strain THY.AP4. Sequences encoding *Cr*ATG8, *At*ATG8D, *At*ATG8G, and *At*ATG8H, as well as the membrane-anchored exodomain of the Arabidopsis receptor IOS1 [61] for negative control, were cloned as preys into pNX32-DEST and transferred into the haploid Mat_alpha_ yeast strain THY.AP5. This strain was also transformed for positive and negative control with the prey plasmids, pNubWt-X-gate and empty pNX32-DEST, respectively. Mating between THY.AP4 and THY.AP5 transformants, and selection of diploids for growth on Synthetic Complete minimum (SC) medium complemented with adenine (A) and histidine (H) was performed as described [32]. Autotrophic growth of yeast cells was determined at 30 °C on SC medium supplemented with 50 µM methionine in the absence of adenine and histidine.

### Live cell imaging

Green and red fluorescence conferred by GFP- and RFP-tagged fusion proteins were detected in optical sections by confocal laser scanning microscopy on an inverted Zeiss LSM 880 microscope, equipped with Argon ion and HeNe lasers as excitation sources. For simultaneous imaging of GFP and RFP, samples were excited at 488 nm for GFP and 561 nm for RFP. Confocal images were processed using the Zeiss ZEN 2 software.

### Transmission electron microscopy

Cells from Wt and transgenic Chlamydomonas lines were cultured in liquid medium until the exponential growth phase (2 × 10^6^ cells/ml) was reached under a 12 h light/12 h dark cycle. Rapamycin was added to 0.5 µM final concentrations at the beginning of the light period. After 4, 8, 12, and 24 h of incubation, cells were collected, fixed in a mixture of cacodylate buffer 0.1 M and glutaraldehyde 2.5%, and stored at 4 °C. Cells where rinsed with buffer then postfixed in 1% osmium tetroxide in cacodylate buffer (0.1 M). The final cell pellets were washed in water, dehydrated in acetone, and embedded in epoxy resin. Uranium- and lead citrate-contrasted thin Sects. (80 nm) were analyzed in a JEOL JEM-1400 120 kV transmission electron microscope. Images were acquired with an 11 MegaPixel SIS Morada CCD camera (Olympus).

### Flow cytometry

Analyses by flow cytometry were performed on a SP6800 spectral cytometer (SONY Biotechnologies). Chlamydomonas cells were labeled with 7.5 μM CFSE (Life technologies; 2 × 10^7^ cells/ml) for 20 min at 37 °C, then washed once in TAP medium and resuspended in appropriate amounts of fresh medium to reach final concentrations of 1 × 10^6^ cells/ml. CFSE-labeled cells were then treated with 0.004% EtOH and 0.001% Tween, or with rapamycin at 0.5 μM. Assessing the beginning of the light period as time point 0, aliquots were taken from the cultures 4, 8, 12, and 24 h later. For each time point, at least 80,000 cells were collected and analyzed for (i) size and overall complexity (FSC and SSC parameters), (ii) chlorophyll content (natural autofluorescence collected after excitation with the 488 and 405 nm laser lines), and (iii) proliferation based on CFSE dilution. Data from single cells were then analyzed with the Kaluza software (Beckman coulter). The dynamics of parameter changes were estimated by measuring the values collected at TN reported to the basal values at the beginning of the kinetics (T0).

### Supplementary Information

**Additional file 1: Table S1.** *P. parasitica* isolates analyzed for AVH195. **Table S2.** Primers used in this study.

**Additional file 2: Fig. S1.** Protein sequence alignment of AVR3b and AVH153 from *P. infestans* and *P. parasitica*, respectively. Aligned are the proteins XP_002997848 (AVR3b; GenBank XM_002997802.1) and L917_11572 (AVH153; GenBank ETL89511.1). The signal peptide sequences for secretion are shaded in blue and the RxLR-EER motifs in red. Amino acid alignments were performed with Clustal Omega and edited with Boxshade. Shading indicates blocks of identical (black) or similar (grey) amino acids. Related to Fig. 2. **Fig. S2.** Simultaneous transient expression of the cell death inducers AVH153, AvrPto and BAX with either the empty vector control (EV) or AVH195. The analysis emphasizes that AVH195 must be present in the plant cell prior to the application of a cell death inducer in order to exert its cell death suppressive effect. It also shows that the agrobacteria used in Fig. 2A for the second infiltration after AVH195 expression are fully efficient in cell death induction. Related to Fig. 2. **Fig. S3.** Phylogenetic relationships of ATG8 sequences from *C. reinhardtii*, tomato (*pSolxxgxxxxxx*) and *A. thaliana* (*Ath-ATG8A-I*). The tree was constructed using the Maximum Likelihood (ML) method based on the LG model with a gamma rate of heterogeneity [62]. Three clades (I-III) were defined, in agreement with other reports [29]. **Fig. S4.** Single optical sections from the maximum projection images in Fig. 4C. The signals of the RFP-tagged AVH195 variants and AtATG8H are shown in the first column, the signals of GFP-tagged INT1 in the second. Channel overlay images are shown in the third column, denoting squares, the details of which are magnified in the fourth column. Changes in subcellular localization were analyzed in control cells (upper three rows) and after stimulation of autophagy with AZD8099 (lower three rows). Relative fluorescence intensity plots of GFP and RFP signals, shown in the two columns, were acquired in the corresponding detailed overlay panels along the lines from a to b and from c to d. Bars represent 10 µm. **Fig. S5.** Parameters for life and death of *Chlamydomonas reinhardtii* Wt and transformant cell lines, as analyzed by flow cytometry. Chlamydomonas cultures from WT and transformed cell lines were analyzed over a 24-h period on an SP6800 spectrocytometer (SONY Biotechnologies). For each time point, at least 80,000 cells were analyzed for the following parameters: Forward light scatter (FSC), side light scatter (SSC), autofluorescence of cells as an indicator of chlorophyll content, DAPI staining to assess cell death, and CFSE distribution in daughter cells, shown here as CFSE^-1^ to emphasize cell proliferation. With the exception of DAPI staining, the left graphs represent the median values of fluorescence intensities (arbitrary units) over a 24-h period, and the right graphs show the distribution of fluorescence intensities within the indicated population of cells collected at the 8-h time point. The left graph for DAPI staining shows the proportion of cells stained positive with DAPI over a 24-h period, and the right graph shows a magnification of the distribution of fluorescence intensities within the population of DAPI-stained cells collected at the 8-h time point. **Fig. S6.** Representative view of Chlamydomonas cells from the wild-type and transgenic lines expressing *AVH195*, as analyzed by TEM. Representative view of Chlamydomonas cells from the wild-type and transgenic lines expressing *AVH195*, as analyzed by TEM. Micrographs show untreated cells, or cells that were incubated with 0.5 µM rapamycin for 4 h, 8 h, 12 h, and 24 h. Bars represent 10 µm. Related to Fig. 5. **Fig. S7.** Overview screen of *Hpa* infection sites on Arabidopsis leaves. Micrographs of 15 trypan blue-stained infection sites on leaves from each the Wt and the transgenic AVH195-expressing lines OE6 and OE9, 24 h after inoculation. Developing hyphae grow overall faster in tissue of the transgenic lines. Bars represent 100 µm.

**Additional file 3.** Original blots.

## Data Availability

The datasets used and/or analyzed during the current study are available from the corresponding author on reasonable request.

## Abbreviations

aa: Amino acid
AIM: Atg8-Interacting Motif
ATG8: Autophagy-related protein 8
Avr: Avirulence
BAX: Bcl-2-Associated X
CaMV: Cauliflower Mosaic Virus
CFSE: 5-,6-Carboxyfluorescein succinimidyl ester
CLSM: Confocal laser scanning microscopy
CRN: Crinkler and necrosis
DAPI: 4′,6-Diamidino-2-phenylindole
FSC: Forward light scattering
GFP: Green fluorescent protein
HMP1: Haustorium-Specific Membrane Protein 1
hpi: Hours post inoculation
INT1: Inositol Transporter 1
LIR: LC3-interacting region
mbSUS: Mating-based split-ubiquitin system
NPP1: Necrosis-inducing *Phytophthora* Protein 1
PSSM: Position-specific scoring matrix
Pst: *Pseudomonas syringae* Pv *tomato*
RFP: Red fluorescent protein
SSC: Side light scattering
TEM: Transmission electron microscopy
Wt: Wild-type
