# Supplementary material for: The Phytophthora parasitica effector AVH195 interacts with ATG8, attenuates host autophagy, and promotes biotrophic infection
Source: BMC Biol. 2024 Apr 29;22:100. doi: 10.1186/s12915-024-01899-w (PMC11057187; doi:10.1186/s12915-024-01899-w)
Supplement: Supplementary file 1 — Additional file 1: Table S1. P. parasitica isolates analyzed for AVH195. Table S2. Primers used in this study. [file 12915_2024_1899_MOESM1_ESM.docx]

**Table S1.** *P. parasitica* isolates analyzed for AVH195.

| **Isolate** | **Bioproject** | **Host** | **Origin** | **Nucleotide Identity (%)** |
| --- | --- | --- | --- | --- |
| INRA_PP310 | PRJNA259235 | Broad range | Australia | - |
| IAC_01/95* | PRJNA205153 | Citrus | Brazil | 99.8 |
| P1569* | PRJNA181332 | Citrus | USA | 99.7 |
| P10297* | PRJNA181331 | Diffenbachia | USA | 100 |
| Chvinca* | PRJNA205156 | Periwinkle | USA | 100 |
| AM587* | PRJNA294216 | Tobacco | China | 98.5 |
| AM588* | PRJNA294216 | Tobacco | China | 99.8 |
| CJ01A1* | PRJNA181330 | Tobacco | USA | 99.7 |
| CJ02B3* | PRJNA205154 | Tobacco | USA | 99.5 |
| CJ05E6* | PRJNA205155 | Tobacco | USA | 99.8 |
| 182 | This work | Tobacco | USA | 99.7 |
| 733 | This work | Tobacco | USA | 99.8 |
| 329 | This work | Tobacco | Greece | 99.8 |
| 397 | This work | Tobacco | USA | 99.7 |
| 721 | This work | Tomato | France | 100 |
| 149 | This work | Tomato | Spain | 100 |
| P1976* | PRJNA181333 | Tomato | USA | 99.8 |
| H2 | This work | Vanilla | French Polynesia | 100 |
| 138 | This work | Tomato | Lebanon | 100 |

Isolates with available genome sequences at Genbank are indicated with an asterisk (*).

**Table S2.** Primers used in this study.

| **Name** | **Organism** | **Sequence 5'3'** | **Comment** |
| --- | --- | --- | --- |
| attB1_Avh195_F | *P. parasitica* | GGGGACAAGTTTGTACAAAAAAGCAGGCTTCACCATGCTATCGGCCTATGAACAA | Gateway cloning |
| attB2_Avh195_R | *P. parasitica* | GGGGACCACTTTGTACAAGAAAGCTGGGTCTCACAAGGCGCTAGCCGCGTG | Gateway cloning |
| 195_AIM_S1_mut_F | *P. parasitica* | GAAAAACGGGAAAAGCGCTGATGACGCCTTCGACCGCTGG | Site-directed mutagenesis |
| 195_AIM_S1_mut_R | *P. parasitica* | CCAGCGGTCGAAGGCGTCATCAGCGCTTTTCCCGTTTTTC | Site-directed mutagenesis |
| 195_AIM_S2_mut_F | *P. parasitica* | CATCTTCGACCGCGCGATTCGAGCCGATAAGTCACCG | Site-directed mutagenesis |
| 195_AIM_S2_mut_R | *P. parasitica* | CGGTGACTTATCGGCTCGAATCGCGCGGTCGAAGATG | Site-directed mutagenesis |
| 195_AIM_S3_mut_F | *P. parasitica* | CAATCAGACCGATTGCGCGCGAAGCCGGACTGACAGAG | Site-directed mutagenesis |
| 195_AIM_S3_mut_R | *P. parasitica* | CTCTGTCAGTCCGGCTTCGCGCGCAATCGGTCTGATTG | Site-directed mutagenesis |
| attB1_CrATG8_F | *C. reinhardtii* | GGGGACAAGTTTGTACAAAAAAGCAGGCTTCACCATGGTTGGCTCCCGACCCCCGAC | Gateway cloning |
| attB2_CrATG8_R | *C. reinhardtii* | GGGGACCACTTTGTACAAGAAAGCTGGGTCTCACAACGCCAGCTCCTCCACA | Gateway cloning |
| pChlamy3_intron_F | *C. reinhardtii* | TGCTTGCAGATTTGACTTGC | Genoytping |
| pChlamy3_spliced_F | *C. reinhardtii* | TAAAATGGCCAGGAGATTCG | Genoytping |
| pChlamy3_RBCS2 3'UTR_R | *C. reinhardtii* | TACCGCTTCAGCACTTGAGA | Genoytping |
| pChlamy3_5'UTR_F | *C. reinhardtii* | GATAAACCGGCCAGGGGGCC | Genoytping |
| pChlamy3_3'UTR_R | *C. reinhardtii* | CAGCAAAAGGTAGGGCGGGC | Genoytping |
| AT5G11770 _F | *A.thaliana* | GAAGTTGTGCCAATGGAGGT | qPCR |
| AT5G11770 _R | *A.thaliana* | CCACCAATGCAAGAAATCCT | qPCR |
| AT5G62050 _F | *A.thaliana* | AACAGGACTCAGCGATGTTG | qPCR |
| AT5G62050_ R | *A.thaliana* | TACCTGATCTGCCTCCACCT | qPCR |
| qUBC_F1 | *P. parasitica* | CCACTTAGAGCACGCTAGGA | qPCR |
| qUBC_R1 | *P. parasitica* | TACCGACTGTCCTTCGTTCA | qPCR |
| qWS41_F1 | *P. parasitica* | TTCAAGTCCAGTGAGATCGG | qPCR |
| qWS41_R1 | *P. parasitica* | TTGTGTCTTTGTGTGATGCG | qPCR |
| q195_F10 | *P. parasitica* | AGGCAAGCAGCCAAAAAC | qPCR |
| q195_R10 | *P. parasitica* | CGGCACGAAGTTGATACTCTG | qPCR |
| q195_F2 | *P. parasitica* | CTTCGTGCAATGCTCTATCG | qPCR |
| q195_R2 | *P. parasitica* | CAGACGTATCTCCGGTTTCAG | qPCR |
| qNPP1_F | *P. parasitica* | CCCCAAATGAACGTCCTTAC | qPCR |
| qNPP1_R | *P. parasitica* | TGAACTTGACACCAGCCTTC | qPCR |
| qHMP1_F | *P. parasitica* | GATCGGTGAGACCATTTTCG | qPCR |
| qHMP1_R | *P. parasitica* | TGTTGAGGAACGTGTCAAGC | qPCR |
| qCre195_1_F | *C. reinhardtii* | ATCTTCGACCGCTGGATTC | qPCR |
| qCre195_1_R | *C. reinhardtii* | TGGTCTCCAGGTTCATGTTG | qPCR |
| CBLP_F | *C. reinhardtii* | GCCACACCGAGTGGGTGTCGTGCG | qPCR |
| CBLP_R | *C. reinhardtii* | CCTTGCCGCCCGAGGCGCACAGCG | qPCR |
| RBCS2_F | *C. reinhardtii* | ATACTGCTCTCAAGTGCTGAAGCG | qPCR |
| RBCS2_R | *C. reinhardtii* | AAAGACTGATCAGCACGAAACGG | qPCR |
