# Supplementary material for: The Phytophthora parasitica effector AVH195 interacts with ATG8, attenuates host autophagy, and promotes biotrophic infection
Source: BMC Biol. 2024 Apr 29;22:100. doi: 10.1186/s12915-024-01899-w (PMC11057187; doi:10.1186/s12915-024-01899-w)
Supplement: Supplementary file 2 — Additional file 2: Fig. S1. Protein sequence alignment of AVR3b and AVH153 from P. infestans and P. parasitica, respectively. Aligned are the proteins XP_002997848 (AVR3b; GenBank XM_002997802.1) and L917_11572 (AVH153; GenBank ETL89511.1). The signal peptide sequences for secretion are shaded in blue and the RxLR-EER motifs in red. Amino acid alignments were performed with Clustal Omega and edited with Boxshade. Shading indicates blocks of identical (black) or similar (grey) amino acids. Related to Fig. 2. Fig. S2. Simultaneous transient expression of the cell death inducers AVH153, AvrPto and BAX with either the empty vector control (EV) or AVH195. The analysis emphasizes that AVH195 must be present in the plant cell prior to the application of a cell death inducer in order to exert its cell death suppressive effect. It also shows that the agrobacteria used in Fig. 2A for the second infiltration after AVH195 expression are fully efficient in cell death induction. Related to Fig. 2. Fig. S3. Phylogenetic relationships of ATG8 sequences from C. reinhardtii, tomato (pSolxxgxxxxxx) and A. thaliana (Ath-ATG8A-I). The tree was constructed using the Maximum Likelihood (ML) method based on the LG model with a gamma rate of heterogeneity [62]. Three clades (I-III) were defined, in agreement with other reports [29]. Fig. S4. Single optical sections from the maximum projection images in Fig. 4C. The signals of the RFP-tagged AVH195 variants and AtATG8H are shown in the first column, the signals of GFP-tagged INT1 in the second. Channel overlay images are shown in the third column, denoting squares, the details of which are magnified in the fourth column. Changes in subcellular localization were analyzed in control cells (upper three rows) and after stimulation of autophagy with AZD8099 (lower three rows). Relative fluorescence intensity plots of GFP and RFP signals, shown in the two columns, were acquired in the corresponding detailed overlay panels along the lines from a to [file 12915_2024_1899_MOESM2_ESM.pdf]

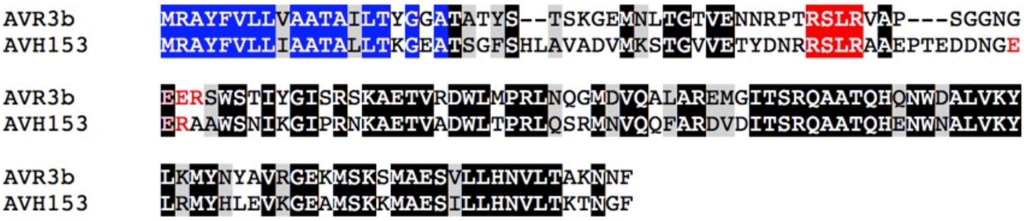

Figure S1. Protein sequence alignment of AVR3b and AVH153 from *P. infestans* and *P. parasitica*, respectively.

Aligned are the proteins XP_002997848 (AVR3b; GenBank XM_002997802.1) and L917_11572 (AVH153; GenBank ETL89511.1). The signal peptide sequences for secretion are shaded in blue and the RxLR-EER motifs in red. Amino acid alignments were performed with Clustal Omega and edited with Boxshade. Shading indicates blocks of identical (black) or similar (grey) amino acids. Related to Figure 2.

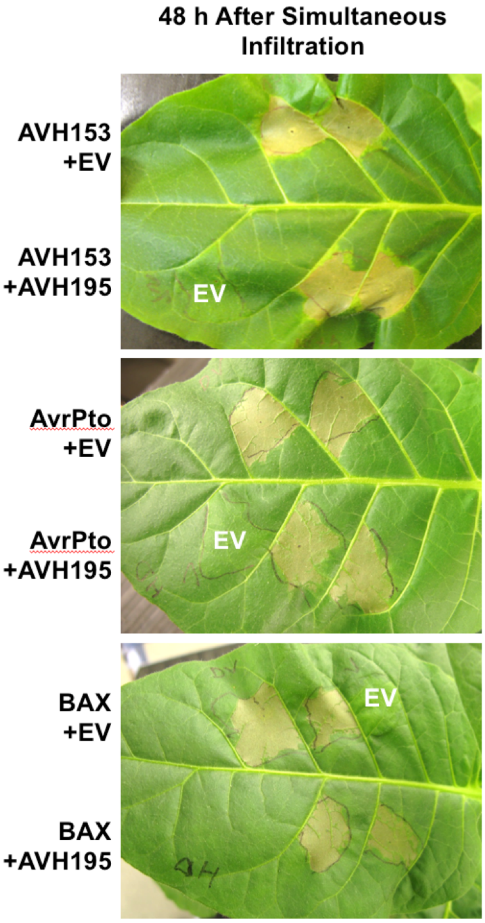

Figure S2. Simultaneous transient expression of the cell death inducers AVH153, AvrPto and BAX with either the empty vector control (EV) or AVH195.
The analysis emphasizes that AVH195 must be present in the plant cell prior to the application of a cell death inducer in order to exert its cell death suppressive effect. It also shows that the agrobacteria used in Figure 2A for the second infiltration after AVH195 expression are fully efficient in cell death induction. Related to Figure 2.

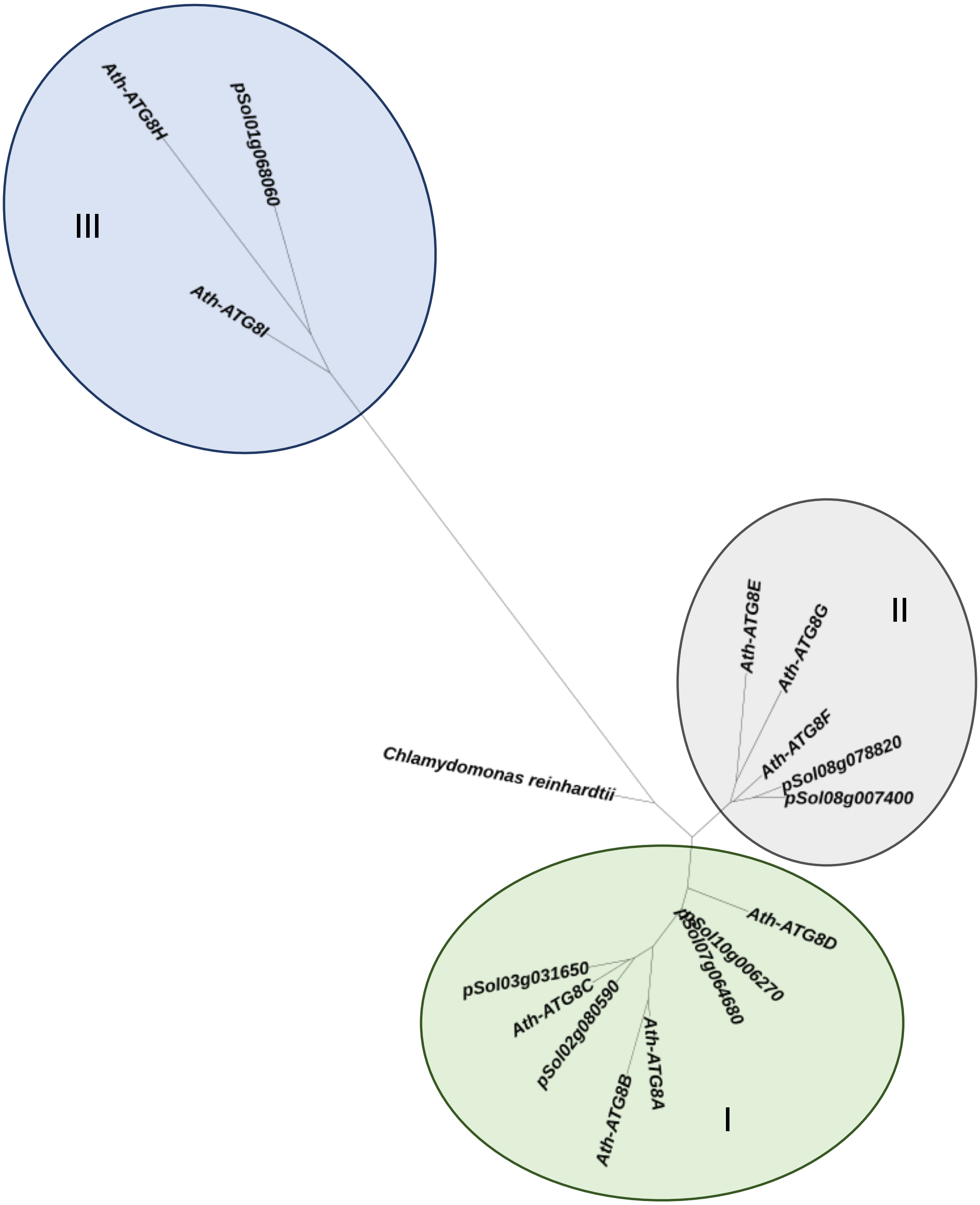

Figure S3. Phylogenetic relationships of ATG8 sequences from *C. reinhardtii*, tomato (*pSolxxgxxxxxx*) and *A. thaliana (Ath-ATG8A-I)*.
The tree was constructed using the Maximum Likelihood (ML) method based on the LG model with a gamma rate of heterogeneity [62]. Three clades (I-III) were defined, in agreement with other reports [29].

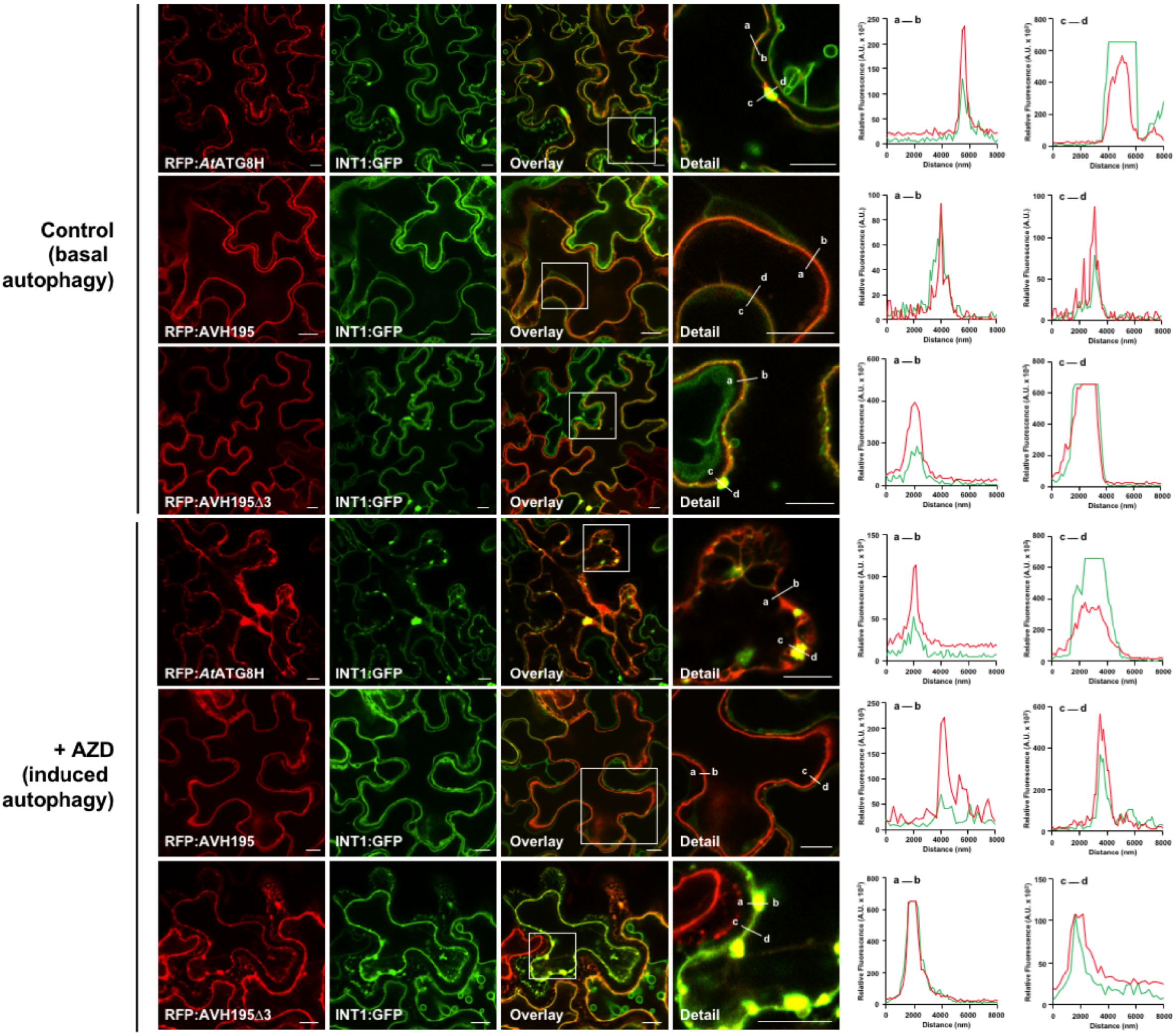

Figure S4. Single optical sections from the maximum projection images in Figure 4C.
The signals of the RFP-tagged AVH195 variants and AtATG8H are shown in the first column, the signals of GFP-tagged INT1 in the second. Channel overlay images are shown in the third column, denoting squares, the details of which are magnified in the fourth column. Changes in subcellular localization were analyzed in control cells (upper three rows) and after stimulation of autophagy with AZD8055 (lower three rows). Relative fluorescence intensity plots of GFP and RFP signals, shown in the two columns, were acquired in the corresponding detailed overlay panels along the lines from a to b and from c to d. Bars represent 10 µm.

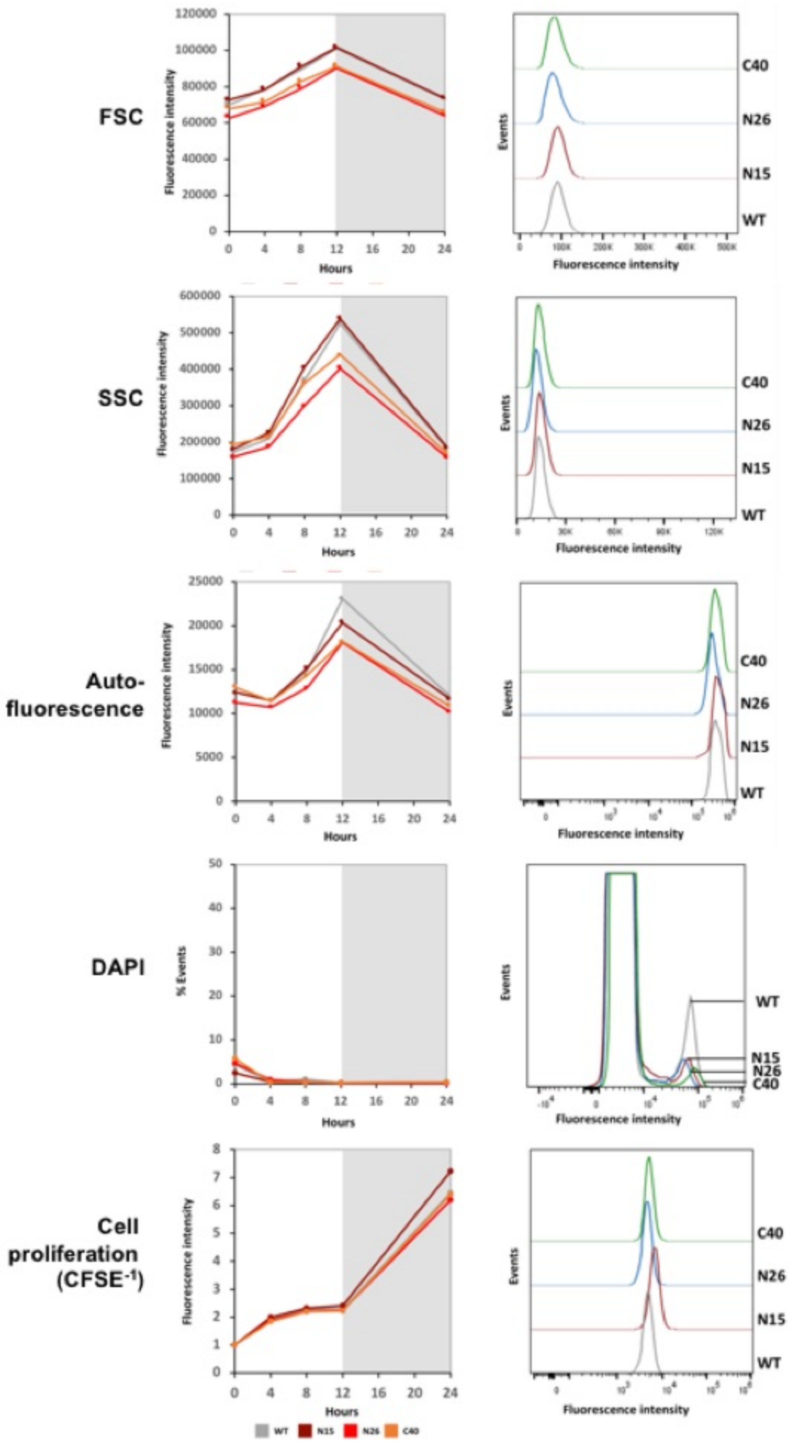

Figure S5. Parameters for life and death of *Chlamydomonas reinhardtii* Wt and transformant cell lines, as analyzed by flow cytometry.

Chlamydomonas cultures from WT and transformed cell lines were analyzed over a 24-hour period on an SP6800 spectrocytometer (SONY Biotechnologies). For each time point, at least 80,000 cells were analyzed for the following parameters: Forward light scatter (FSC), side light scatter (SSC), autofluorescence of cells as an indicator of chlorophyll content, DAPI staining to assess cell death, and CFSE distribution in daughter cells, shown here as $CFSE^{-1}$ to emphasize cell proliferation. With the exception of DAPI staining, the left graphs represent the median values of fluorescence intensities (arbitrary units) over a 24-hour period, and the right graphs show the distribution of fluorescence intensities within the indicated population of cells collected at the 8-hour time point. The left graph for DAPI staining shows the proportion of cells stained positive with DAPI over a 24-hour period, and the right graph shows a magnification of the distribution of fluorescence intensities within the population of DAPI-stained cells collected at the 8-hour time point.

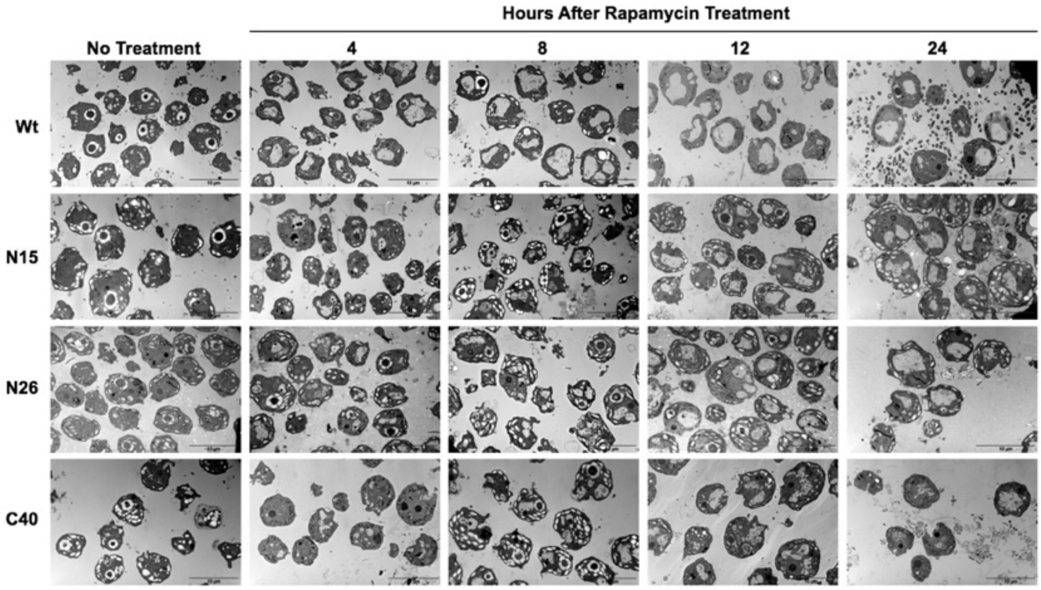

Figure S6. Representative view of Chlamydomonas cells from the wild-type and transgenic lines expressing *AVH195*, as analyzed by TEM. Micrographs show untreated cells, or cells that were incubated with 0.5 µM rapamycin for 4 h, 8 h, 12 h, and 24 h. Bars represent 10 µm. Related to Figure 5.

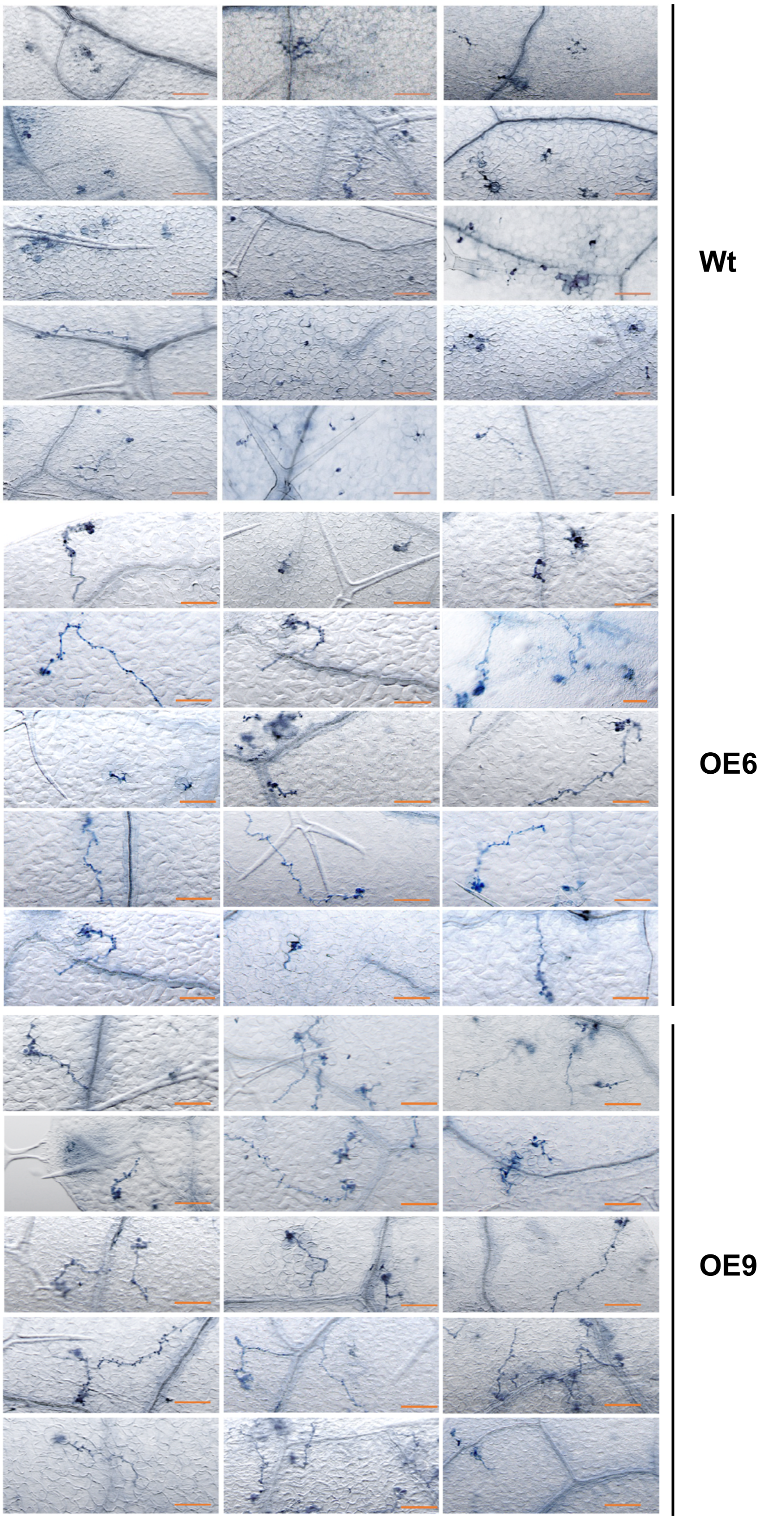

Figure S7. Overview screen of *Hpa* infection sites on Arabidopsis leaves. Micrographs of 15 trypan blue-stained infection sites on leaves from each the Wt and the transgenic *AVH195*-expressing lines OE6 and OE9, 24 h after inoculation. Developing hyphae grow overall faster in tissue of the transgenic lines. Bars represent 100 µm. Related to Figure 6.
