# Supplementary material for: The Phytophthora parasitica effector AVH195 interacts with ATG8, attenuates host autophagy, and promotes biotrophic infection
Source: BMC Biol. 2024 Apr 29;22:100. doi: 10.1186/s12915-024-01899-w (PMC11057187; doi:10.1186/s12915-024-01899-w)
Supplement: Supplementary file 3 — Additional file 3. Original blots. [file 12915_2024_1899_MOESM3_ESM.pdf]

Additional File 3: Uncropped blots Figure 3A

M

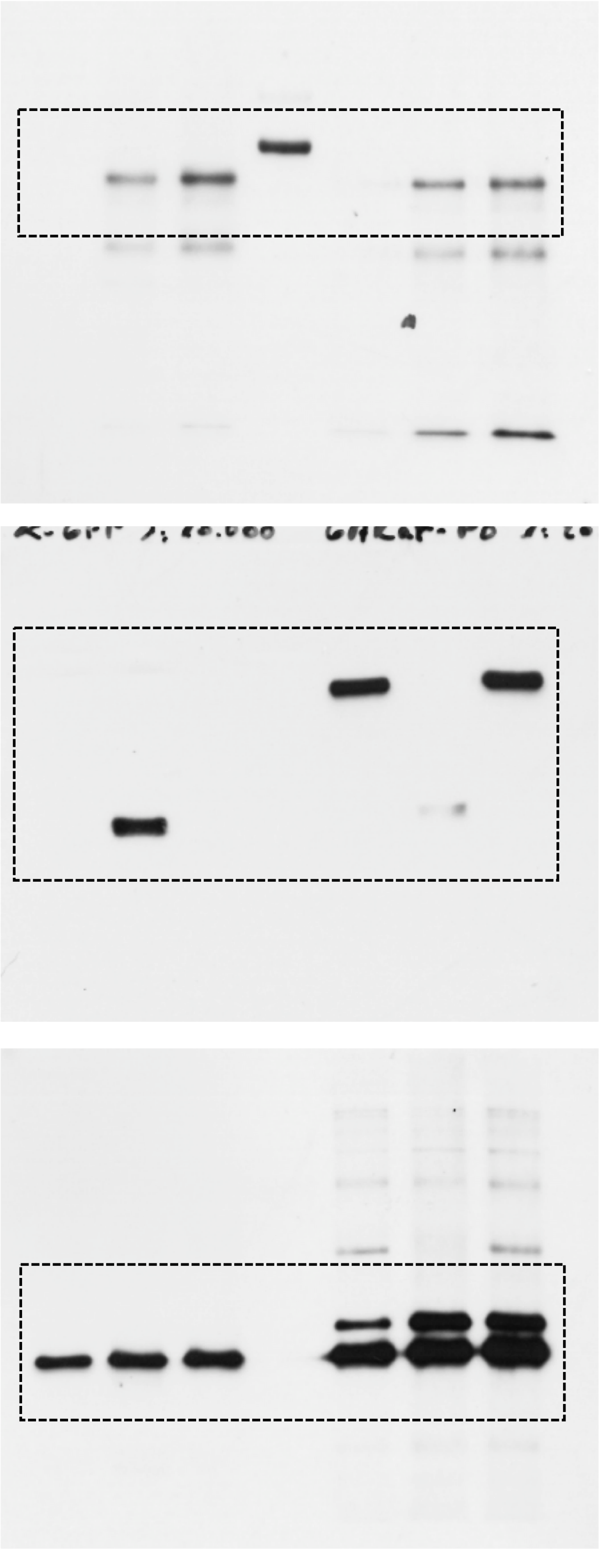

α–ATG8

α–GFP

α–PsbO

Limits of the displayed area in Figure 3A

M Protein marker lane

Additional File 3: Uncropped blots Figure 4B

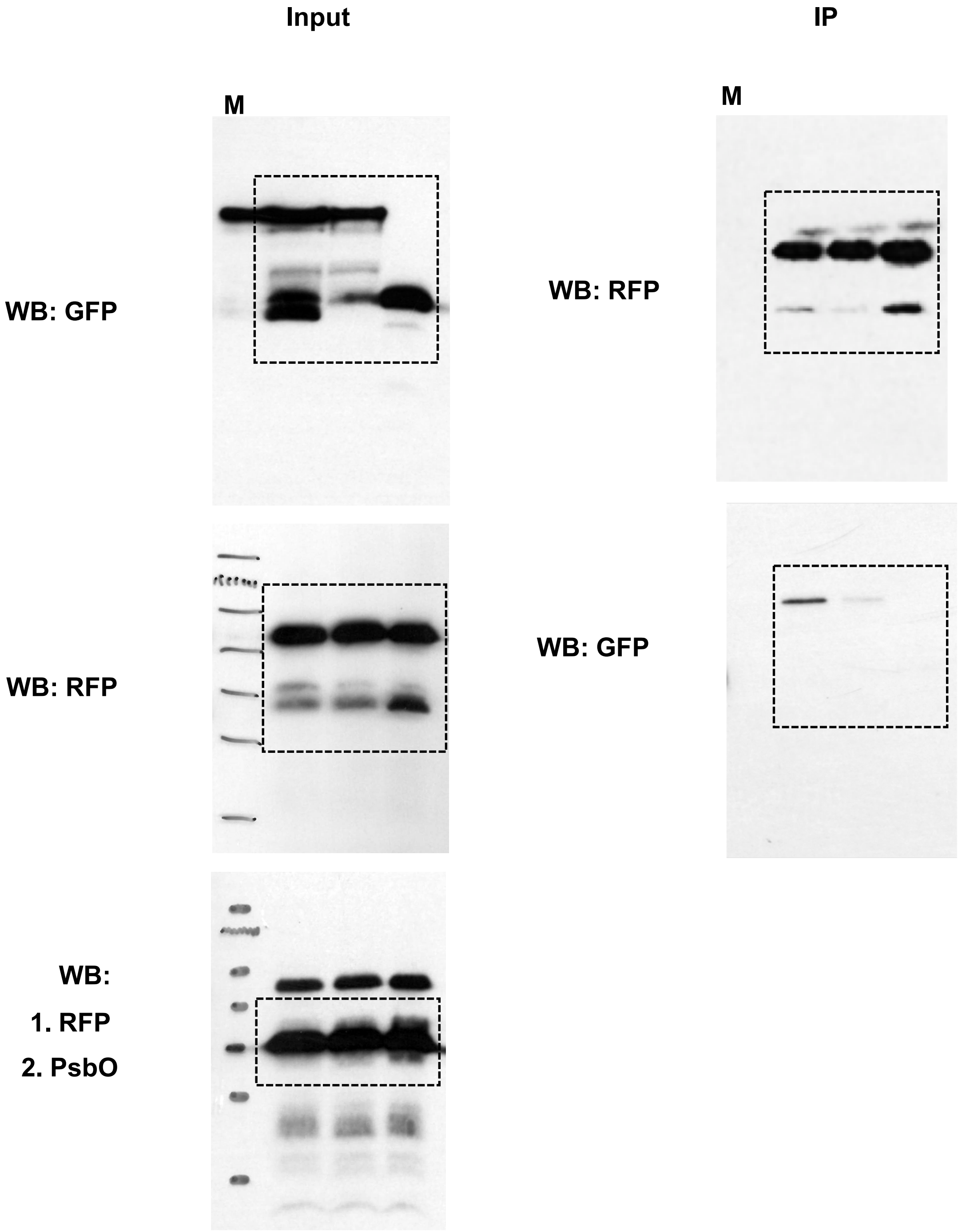

Limits of the displayed area in Figure 4B

M Protein marker lane

Additional File 3: Uncropped blots Figure 6A

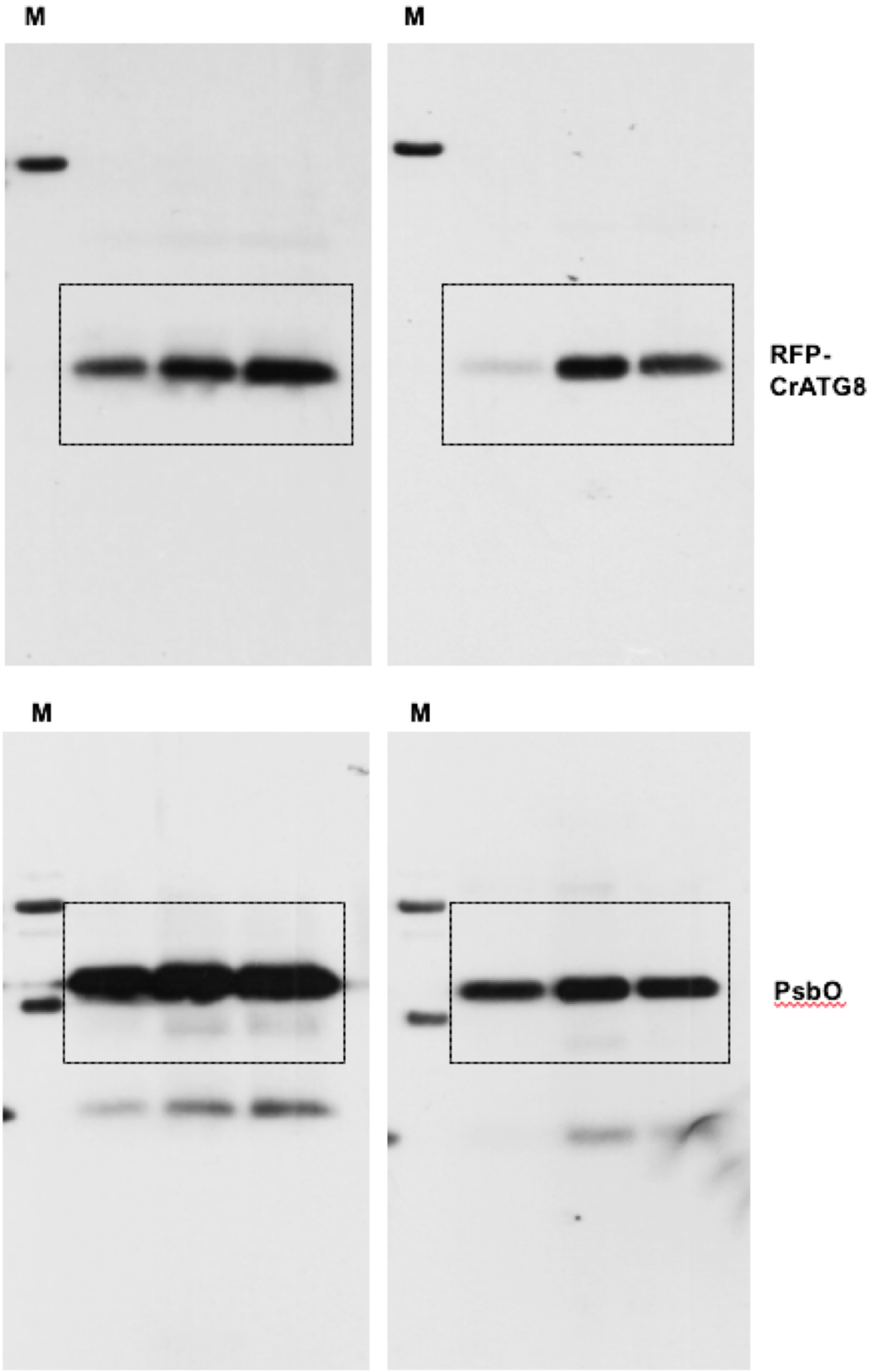

Limits of the displayed area in Figure 6A

M Protein marker lane
